# Combining stereo‐video monitoring and physiological trials to estimate reef fish metabolic demands in the wild

**DOI:** 10.1002/ece3.9084

**Published:** 2022-07-05

**Authors:** Nina M. D. Schiettekatte, Francesca Conte, Beverly French, Simon J. Brandl, Christopher J. Fulton, Alexandre Mercière, Tommy Norin, Sébastien Villéger, Valeriano Parravicini

**Affiliations:** ^1^ PSL Université Paris: EPHE‐UPVD‐CNRS USR 3278 CRIOBE, Université de Perpignan Perpignan France; ^2^ Laboratoire d'Excellence “CORAIL” Perpignan France; ^3^ Hawai'i Institute of Marine Biology University of Hawai'i at Mānoa Hawaii USA; ^4^ Center for Marine Biodiversity and Conservation Scripps Institution of Oceanography, University of California San Diego California USA; ^5^ CESAB‐FRB Montpellier France; ^6^ Department of Marine Science, Marine Science Institute The University of Texas at Austin Port Aransas Texas USA; ^7^ Australian Institute of Marine Science Indian Ocean Marine Research Centre Crawley Western Australia Australia; ^8^ DTU Aqua: National Institute of Aquatic Resources Technical University of Denmark Kgs. Lyngby Denmark; ^9^ MARBEC, Université de Montpellier CNRS, IFREMER, IRD Montpellier France

**Keywords:** activity, activity scope, field metabolic rate, fish, metabolic scaling, metabolism, swimming speed

## Abstract

Organismal metabolic rates (MRs) are the basis of energy and nutrient fluxes through ecosystems. In the marine realm, fishes are some of the most prominent consumers. However, their metabolic demand in the wild (field MR [FMR]) is poorly documented, because it is challenging to measure directly. Here, we introduce a novel approach to estimating the component of FMR associated with voluntary activity (i.e., the field active MR [$\mathrm{AM}R_{\text{field}}]$). Our approach combines laboratory‐based respirometry, swimming speeds, and field‐based stereo‐video systems to estimate the activity of individuals. We exemplify our approach by focusing on six coral reef fish species, for which we quantified standard MR and maximum MR (SMR and MMR, respectively) in the laboratory, and body sizes and swimming speeds in the field. Based on the relationships between MR, body size, and swimming speeds, we estimate that the activity scope (i.e., the ratio between $\mathrm{AM}R_{\text{field}}$ and SMR) varies from 1.2 to 3.2 across species and body sizes. Furthermore, we illustrate that the scaling exponent for $\mathrm{AM}R_{\text{field}}$ varies across species and can substantially exceed the widely assumed value of 0.75 for SMR. Finally, by scaling organismal $\mathrm{AM}R_{\text{field}}$ estimates to the assemblage level, we show the potential effect of this variability on community metabolic demand. Our approach may improve our ability to estimate elemental fluxes mediated by a critically important group of aquatic animals through a non‐destructive, widely applicable technique.

## Graphical abstract

We know little about the metabolic demand of fishes in the wild. We propose a new approach to estimate active field metabolic rates by combining laboratory‐based respirometry and field‐based stereo‐video systems.

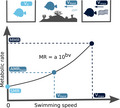

## 1 INTRODUCTION

Anthropogenic stressors, such as climate change, over‐harvesting, and pollution, are affecting biological communities at an unprecedented rate (Halpern et al., 2008; Venter et al., 2016). Scientists and policy‐makers are becoming increasingly concerned that these community impacts will irreversibly alter key ecosystem functions, preventing these natural systems from maintaining indispensable services to humans (Cardinale et al., 2012). In this context, tools to quantify and monitor ecosystem processes are valuable (Tilman et al., 2014). However, while there is a long‐standing tradition in measuring ecological processes in mesocosms and controlled in situ experiments, the assessment of rates of ecological processes in natural conditions is still in its infancy (Reich et al., 2012), especially for marine ecosystems (Brandl, Rasher, et al., 2019).

In coastal marine ecosystems, fishes represent one of the most thoroughly studied, ecologically important, and economically valuable group of consumers (Bozec et al., 2004; Tamayo et al., 2018). Despite the complexity of measuring the contribution of mobile species to ecosystem fluxes (Wilson et al., 2010), several attempts have been made to quantify the contributions of fishes to nutrient and carbon cycling (Brandl, Rasher, et al., 2019; Villéger et al., 2017). These functions are usually quantified at the individual level, which can then be scaled up to community levels through an additive framework (Allgeier et al., 2014; Barneche et al., 2014; Brandl, Tornabene, et al., 2019; Morais & Bellwood, 2019). While there are inherent limitations to this approach, individual‐based modeling currently represents our best means to quantify ecological processes across communities of mobile, aquatic organisms. Nevertheless, the accuracy of these approaches inevitably depends on our capacity to precisely estimate the physiological requirements and expenditures of individuals in their natural environment.

The metabolic rate (MR) of living organisms is an essential determinant of their physiological requirements and therefore represents a crucial parameter to estimate the flow of energy and nutrients in any ecosystem (Allen et al., 2005; Brown et al., 2004). MRs of fishes are generally evaluated through two metrics: (i) standard MR (SMR) (Fry, 1957; Vinberg, 1960), which corresponds to the MR of an inactive and fasting individual (Chabot et al., 2016) and (ii) maximum MR (MMR), which corresponds to the aerobic MR of an animal that is exercising at full capacity (Norin & Clark, 2016). Theory predicts that individual MR increases hypoallometric (sub‐linearly) with body mass according to a power function with a scaling exponent of approximately 0.75 (Brown et al., 2004; Gillooly et al., 2001; West et al., 1997). While laboratory measurements of the SMR of resting fishes have both confirmed a scaling exponent close to 0.75 (Barneche et al., 2014; Clarke & Johnston, 1999) and rejected it (Bokma, 2004; Killen et al., 2016), an 0.75 scaling exponent has been used to estimate community‐level MRs (Cheung et al., 2013; Deutsch et al., 2015; Holt & Jørgensen, 2015).

Knowledge of SMR and MMR allows for the calculation of a fish's aerobic scope, which is the ratio between MMR and SMR and represents the capacity to elevate MR above maintenance to support energetically demanding tasks such as physical activity and digestion (Clark et al., 2013). Within species, aerobic scope tends to increase with body mass regardless of being expressed in absolute (MMR minus SMR) or factorial (MMR divided by SMR) values (Halsey et al., 2018), as the scaling exponent of MMR is often observed to be higher than that of SMR (Glazier, 2005; Killen et al., 2007). Both SMR and MMR can be estimated relatively accurately in the laboratory through measurements of oxygen uptake rates (Clark et al., 2013; Svendsen et al., 2016). However, animals in the wild rarely reside at SMR or exercise maximally. Thus, calculations of energy expenditures in wild fishes are hamstrung by our inability to accurately estimate MRs in fishes that pursue their normal, daily activities in their natural environment.

The field MR (FMR) represents the average MR of an individual in the wild (Chung et al., 2019; Nagy, 2005) and lies somewhere between SMR and MMR (Nagy, 2005). On average, free‐living fishes in their natural habitats will only exploit a given proportion of their aerobic scope and, in non‐sedentary fishes, physical activity will be a major component of FMR (Chung et al., 2019). Thus, the factorial scope for activity (FSA), which corresponds to the ratio between the component of FMR related to physical activity (the $\mathrm{AM}R_{\text{field}}$) and the SMR, is a better reflection of energy expenditure in the wild than the aerobic scope (Chung et al., 2019), bearing in mind that internal homeostatic processes such as digestion and reproduction also incur an energetic cost as part of the full FMR. In terrestrial vertebrates, where the doubly labeled water technique has allowed for widespread quantification of FMR (Webster & Weathers, 1989), the metabolic scaling exponent of FMR tends to be higher than that of SMR (~0.8; Nagy, 2005). While the metabolic scaling exponent of MMR in fishes approximate or exceed 0.8 as well, the scaling of FMR or $\mathrm{AM}R_{\text{field}}$ remains poorly documented (Norin & Clark, 2016). Understanding how MR scales with body mass in the wild is fundamentally important for fisheries (e.g., stock assessments) and predictions of the effects of climate change, as the metabolic scaling exponent is an integral part of growth models used to forecast the size of fishes at both current and future temperatures (Von Bertalanffy, 1957; Cheung et al., 2013; Deutsch et al., 2015; Marshall & White, 2019).

Since FMR is challenging to measure for water‐breathing animals in the aquatic environment (Treberg et al., 2016), it has only been estimated for a small number of fishes (e.g., Chung et al., 2019; Cruz‐Font et al., 2016; Lucas et al., 1993; Murchie et al., 2011). These estimates are largely derived from biotelemetry approaches that rely on accelerometry tags and heart rate measurements calibrated with rates of oxygen uptake in the laboratory (Gräns et al., 2009; Treberg et al., 2016). A major limitation of biotelemetry is that its application is limited to large individuals as it requires surgical attachment or implantation of tags (Gräns et al., 2009). More recently, FMR has been estimated from the isotopic composition of carbon in fish otoliths (Chung et al., 2019). However, this approach relies on destructive sampling and the generality of the undoubtedly promising results are yet to be applied across a broad range of species. Thus, non‐invasive methods to estimate FMR on many co‐occurring fish species in the field are needed to better understand the contributions of fishes to ecosystem functioning.

Here, we propose a new approach to estimating a major component of the FMR, the $\mathrm{AM}R_{\text{field}}$. Specifically, we estimated the SMR and MMR of six reef fish species using traditional respirometry techniques in the laboratory, and then quantified in situ swimming speeds of the same species using underwater stereo‐video systems. This permitted us to derive $\mathrm{AM}R_{\text{field}}$ and the FSA on the basis of the theoretical relationship between MR and swimming speed, and to assess the mass‐scaling exponents of $\mathrm{AM}R_{\text{field}}$ for each species. By combining our results with underwater visual census data of fish size and abundance on reefs around Mo′orea, French Polynesia, we also estimate assemblage‐level SMR and $\mathrm{AM}R_{\text{field}}$. In doing so, we demonstrate the potential applicability of our approach to tackle questions across fields of organismal, community, and ecosystem ecology in the marine biome.

## 2 METHODS

### 2.1 Theory

**FIGURE 1:**
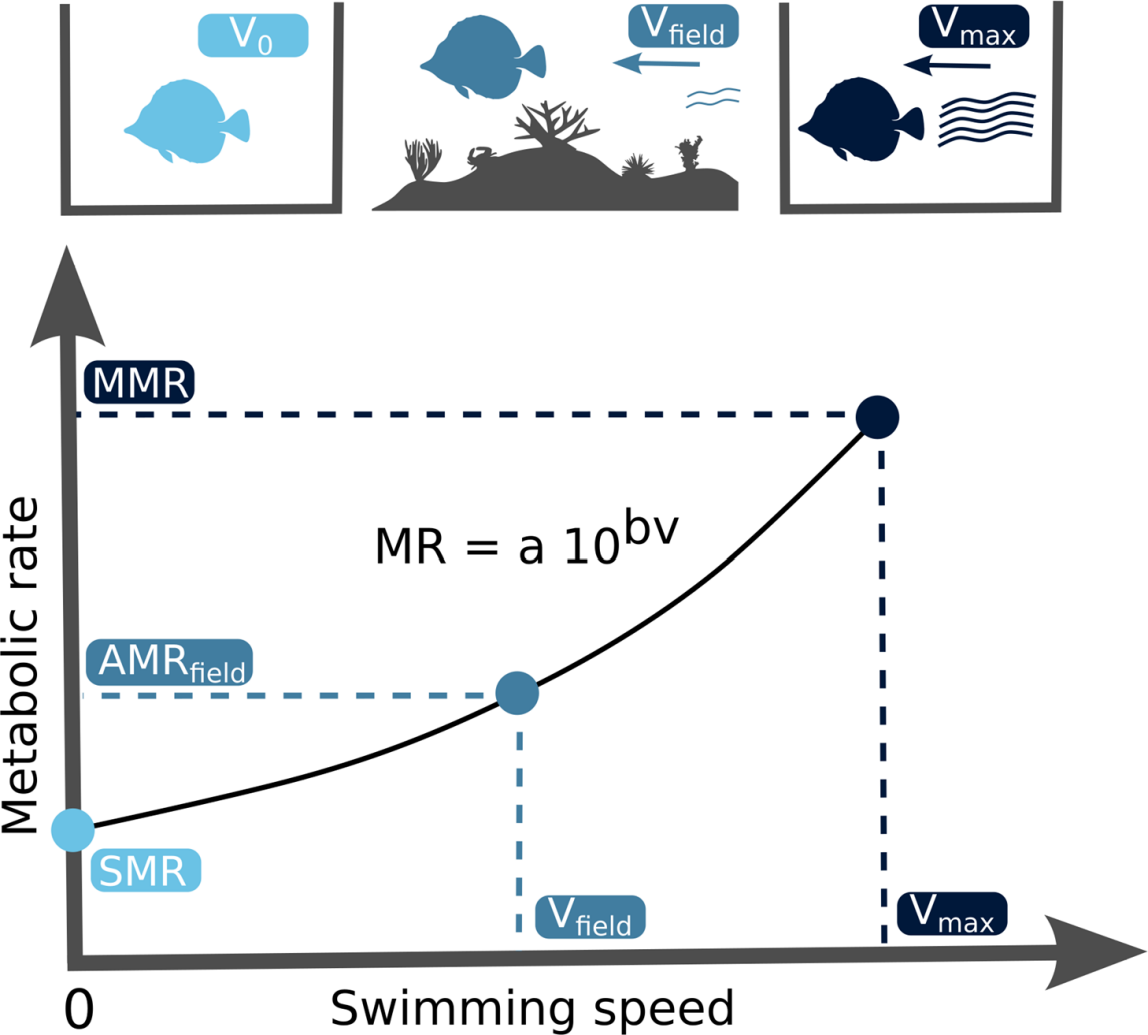
Definition of fish metabolic rates along with the swimming speed range. SMR is the standard metabolic rate at swimming speed (v) zero. $\mathrm{AM}R_{\text{field}}$ is field active metabolic rate at average swimming speed in the field ($v_{\text{field}}$). MMR is maximum metabolic rate, assumed to be reached at maximum swimming speed ($v_{\max}$)

Our approach to quantifying the $\mathrm{AM}R_{\text{field}}$ is based on the relationship between swimming speed (v) and MR (Binning et al., 2013; Norin & Clark, 2016; Torres & Childress, 1983) (Figure 1). We assume that MRs vary predictably with swimming speed following a traditional power function, which can be adapted to a $\mathrm{lo}g_{10}$ transformed form (Brett, 1964; Korsmeyer et al., 2002):

(1) $$\mathrm{MR}=a10^{\mathrm{bv}}$$

(2) $$\mathrm{lo}g_{10}\left(\mathrm{MR}\right)=\mathrm{lo}g_{10}\left(a\right)+\mathrm{bv},$$

We further assume that (1) the SMR represents the MR of an individual when its swimming speed is zero and (2) the MMR represents the oxygen uptake rate of individuals at their maximum swimming speed ($v_{\max}$; Figure 1). The previous equation can thus be rewritten as:

(3) $$\mathrm{lo}g_{10}\left(\mathrm{MR}\right)=\mathrm{lo}g_{10}\left(\mathrm{SMR}\right)+\frac{\mathrm{lo}g_{10}\left(\mathrm{MMR}\right)-\mathrm{lo}g_{10}\left(\mathrm{SMR}\right)}{v_{\max}}v,$$

and $\mathrm{AM}R_{\text{field}}$ can then be estimated using the average swimming speed in the field ($v_{\text{field}}$):

(4) $$\mathrm{lo}g_{10}\left(\mathrm{AM}R_{\text{field}}\right)=\mathrm{lo}g_{10}\left(\mathrm{SMR}\right)+\frac{\mathrm{lo}g_{10}\left(\mathrm{MMR}\right)-\mathrm{lo}g_{10}\left(\mathrm{SMR}\right)}{v_{\max}}v_{\text{field}},$$

The FSA is computed by dividing a fish's $\mathrm{AM}R_{\text{field}}$ by their SMR. We note that the FSA tends to be calculated over a 24 period, which means that we have to consider the amount of time spent resting. If a fish is resting for a certain amount of hours (t) a day and the MR at resting equals the SMR, the FSA can be estimated as follows:

(5) $$\mathrm{FSA}=\frac{\left(24-t\right)\mathrm{AM}R_{\text{field}}+\text{tSMR}}{24\mathrm{SMR}}.$$

Thus, on the basis of known SMR and MMR estimates along with the $v_{\max}$ of individuals, $\mathrm{AM}R_{\text{field}}$ of a species can be estimated if the average swimming speed in the field ($v_{\text{field}}$) for specific body size is known. For our case study, we estimated SMR and MMR using respirometry in the laboratory, obtained $v_{\max}$ through empirical data available in the literature, and estimated v using stereo‐camera video recordings in the field. We then used these estimates of $\mathrm{AM}R_{\text{field}}$ to estimate the FSA and the metabolic scaling exponent for $\mathrm{AM}R_{\text{field}}$. Finally, to evaluate the impact of assessing assemblage‐level MRs on the basis of a realistic proxy of FMR, $\mathrm{AM}R_{\text{field}}$ (instead of using the more commonly measured SMR as an estimate of minimum energetic requirements), we scaled up our estimates at the assemblage level according to visual census data of fish sizes and abundances on a coral reef in Mo′orea, French Polynesia.

### 2.2 Case study species

We focused on six common reef fish species with varying body sizes and shapes, trophic strategies, and behavioral patterns: *Cephalopholis argus* (family Serranidae), a large, fusiform, sedentary piscivore; *Chaetodon ornatissimus* (family Chaetodontidae), a small‐bodied, laterally compressed, obligate coral feeder; *Ctenochaetus striatus* (family Acanthuridae), a medium‐sized, grazing detritivore; *Naso lituratus* (family Acanthuridae), a large‐bodied, grazing herbivore feeding on macroalgae; *Odonus niger* (family Balistidae), a large‐bodied schooling planktivore; and *Zebrasoma scopas* (family Acanthuridae), a compressed, small‐bodied, grazing herbivore feeding on filamentous algae. All data were collected in Mo′orea, French Polynesia, between March 2018 and February 2019. For respirometry experiments, individuals were collected in the lagoon (depth range 1–6 m) next to Opunohu Bay (17.4928°S, 149.8555°W) with hand nets and clove oil. Immediately after capture, the individuals were transported to aquaria in the laboratory and started an acclimatization and fasting period of a minimum of 48 h. The water temperature of the aquaria was controlled by the ambient sea temperature which fluctuated around 28°C for the duration of the experiments. All protocols related to the capture and handling of fish complied with the ethical standards of the Centre for Island Research and Environmental Observatory (CRIOBE).

### 2.3 Standard and maximum metabolic rates

To quantify SMR and MMR, we conducted intermittent‐closed respirometry experiments (Clark et al., 2013; Steffensen, 1989) at 28 ± 0.5°C on a total of 68 individuals across the six study species with the sample size per species ranging between four and 23 individuals. After an acclimation and fasting period of 48 h in aquaria, the fish were individually transferred to a water‐filled tub at 28°C and sequentially chased by the experimenter until visibly exhausted to elicit MMR (Norin & Clark, 2016). Once the chasing was concluded, each individual was immediately placed in a respirometry chamber submerged in an ambient and temperature‐controlled tank, where they were left for approximately 24 h to reach SMR. The intermittent respirometry cycles consisted of a measurement (closed) period followed by an open period during which the respirometry chambers were flushed with fully aerated water from the ambient tank. Based on previous work (Norin & Clark, 2016), we considered the oxygen uptake rate ($\dot{M}O_{2}$) during the first closed cycle (directly after transferring the fish) to be reflective of the individual's MMR. Depending on fish size, respirometry chambers ranged in volume (including tubes and pumps) from 0.38 to 4.4 L, and measurement and flush periods lasted between 2–9 and 3–5 min, respectively. SMR was calculated as the average of the 10% lowest $\dot{M}O_{2}$ values measured during the entire respirometry trial, after the removal of outliers, while MMR was calculated from the slope of the first measurement period (Chabot et al., 2016).

### 2.4 Field swimming speeds

We used two underwater stereo‐video systems that were placed on the seafloor to record fish movements in the wild. Each video system consisted of two small action cameras (GoPro Hero5 Black), mounted 90 cm from each other at an angle of approximately 6°. This method allows three‐dimensional (3D) measurements (Butail & Paley, 2012; Hughes & Kelly, 1996). To analyze the recorded videos, we used VidSync, an open‐source Mac application providing accurate 3D measurements (Neuswanger et al., 2016), which allows for synchronization, calibration, and analysis of videos. We recorded calibration videos to correct for the nonlinear optical distortion of the images due to camera lenses and underwater housings, and to define the 3D coordinate system (*x*, *y*, *z*) used throughout the analyses. Errors in length measurements through video analysis increase with distance from the cameras (Neuswanger et al., 2016). Thus, for each underwater stereo‐video system, we fitted a linear regression model describing the error in measurements as a function of their distance from the nearest camera, which we used to adjust all measurements of distances and fish lengths (Figure 1). We recorded 20 stationary stereo videos between November 19, 2018 and December 12, 2018. Videos were recorded at 12–14 m depth on the reef slope at the Tiahura long‐term monitoring site in Mo′orea (17°29′00.6″S, 149°54′20.9″W) and at five different time periods: 5:00–7:00, 8:00–10:00, 11:00–13:00, 14:00–16:00, and 17:00–18:00. Each recording lasted for ~1 to 1.5 h. We then took measurements during three 10 min sequences with intervals of 10 min that excluded the first 2 min to account for the presence of divers. We took measurements for all fishes visible in both cameras for 3–5 s during the three 10 min sequences. For each individual, fork length was measured three times from the videos as the straight‐line distance between the fish's head and its tail fork, and three to five consecutive swimming speeds were measured as the distance the fish moved over 3–5 s. Final fish lengths and swimming speeds were then calculated as the mean of the repeated measurements. In total, we recorded lengths and speeds for 634 individuals, with sample sizes per species ranging between 64 and 264 individuals.

### 2.5 Maximum swimming speed

We assumed maximum swimming speeds ($v_{\max}$) from previous work that estimated the swimming speed at which a fish becomes exhausted and stops swimming when it is exposed to regular incremental changes in speed in an experimental flume (Brett, 1964; Fulton, 2007). In the original work of Fulton (2007), the maximum swimming speeds of 192 individuals from five fish families and their corresponding lengths were measured, and these measurements were then used in the present study to relate maximum swimming speed with body size and caudal aspect ratio as a proxy for variations in swimming ability while accounting for the effect of fish family and body shape type. Caudal aspect ratio and body shape values were retrieved from Fishbase (Froese & Pauly, 2018).

### 2.6 Data analysis

We quantified $\mathrm{AM}R_{\text{field}}$ and FSA by combining multiple regression models, that describe the relationships between SMR and MMR with body mass, swimming speed (v), and maximum swimming speed ($v_{\max}$; from Fulton, 2007) with body size. First, we used the respirometry data to fit a relationship between either SMR or MMR and body mass using a Bayesian hierarchical model, while accounting for the co‐variation between MMR and SMR. We define the $\mathrm{lo}g_{10}$ of SMR and MMR to be normally distributed with a mean (μ) and a standard deviation (σ) as follows:

(6) $$\log_{10}\left({\mathrm{MR}}_{i}\right)∼\text{Normal}\left(\mu_{i},\sigma \right),$$

(7) $$\mu_{i}=\left(a+a_{j,k}\right)+\left(b+b_{j,k}\right)\log_{10}\left({\text{weight}}_{i}\right),$$

where i is the individual, j is the species, k is the type of MR (SMR or MMR), a is the global intercept of the regression; $a_{j,k}$ is the effect on the intercept for each species and type of MR, b is the global slope of $\mathrm{lo}g_{10}\left(\text{weight}\right)$, and $b_{j,k}$ is the effect on the slope for each species and type of MR. We obtained the mean intercept and slope per species by summing global‐ and species‐level parameters. We used an informative normal prior for the global slope exponent (i.e., metabolic scaling exponent) with an average of 0.75 and 0.1 as the standard deviation (West et al., 1997). For all other parameters, we used weakly informative priors (Burkner, 2017).

Second, using the data retrieved from the video analyses, we fitted a hierarchical Bayesian regression model for estimating fish swimming speed as a function of body length. We defined the $\mathrm{lo}g_{10}$ transformation of swimming speed to be the student *t*‐distributed with degrees of freedom (ν), mean (μ), and a standard deviation (σ). The student's *t*‐distribution was applied to build a robust regression, as our data includes outliers (Motulsky & Brown, 2006).

(8) $$\log_{10}\left(v_{i}\right)∼\text{Student}\left(\nu ,\mu_{i},\sigma \right),$$

(9) $$\mu_{i}=\left(a+a_{j}\right)+\left(b+b_{j}\right)\log_{10}\left({\text{length}}_{i}\right),$$

where v is the swimming speed, i is the individual, j is the species, a is the global intercept of the regression, $a_{j}$ is the effect on the intercept for each species, b is the global slope, and $b_{j}$ is the effect on the slope of each species. For each species, regression exponents were estimated by summing two effects of the model: the global parameter and the species‐specific effect on the global parameter.

Thirdly, we fitted a similar model to predict maximum swimming speed in function of body length and aspect ratio using data extracted from Fulton (2007), including random effects of the interaction between family and body shape on the intercept and slope of body size.

(10) $$\mathrm{lo}g_{10}\left(\text{maxspee}d_{i}\right)∼\text{Student}\left(\nu ,\mu_{i},\sigma \right),$$

(11) $$\mu_{i}=\left(a+a_{j}\right)+\left(b+b_{j}\right)\mathrm{lo}g_{10}\left(\text{lengt}h_{i}\right)+\mathrm{AR},$$

where i is the individual,j is the interaction of family and body shape, a is the global intercept of the regression, $a_{j}$ is the effect on the intercept for each family and body shape, b is the global slope, $b_{j}$ is the effect on the slope for each family and body shape, and AR is the aspect ratio of the tail. Here, we also applied the Student's *t*‐distribution and used general uninformative priors. We then used this model to estimate the maximum swimming speed of the species included in our study.

### 2.7 FAS, $\mathrm{AM}R_{\text{field}}$, and FSA calculations

We estimated the factorial aerobic scope (FAS), $\mathrm{AM}R_{\text{field}}$, and FSA for the full‐size range of all model species (per cm). To estimate the fish's FAS at each possible length, we first predicted their SMR and MMR by estimating their weight using the published length–weight relationship accessed through FishBase (Froese et al., 2014), and making predictions based on our model parameters. For each iteration of the prediction, we calculated FAS as $\mathrm{FAS}=\frac{\mathrm{MMR}}{\mathrm{SMR}}$ (F. Fry, 1947; Killen et al., 2016). Finally, we summarized the FAS for each species at all sizes by taking means, standard deviations, and 95% credible intervals.

To estimate $\mathrm{AM}R_{\text{field}}$, we needed the SMR, MMR, $v_{\max}$, and $v_{\text{field}}$ ($\mathrm{lo}g_{10}\left(\mathrm{AM}R_{\text{field}}\right)=\mathrm{lo}g_{10}\left(\mathrm{SMR}\right)+\frac{\mathrm{lo}g_{10}\left(\mathrm{MMR}\right)-\mathrm{lo}g_{10}\left(\mathrm{SMR}\right)}{v_{\max}}v_{\text{field}}$). For each length and species, we estimated $v_{\text{field}}$, $v_{\max}$, SMR, and MMR using the above‐mentioned regression models. To incorporate an estimate of uncertainty, we included 1000 iterations for $v_{\text{field}}$. For $v_{\max}$, SMR and MMR we used the median of the predicted values in this step.

Once we determined $\mathrm{AM}R_{\text{field}}$, we calculated FSA with the following equation:

(12) $$\mathrm{FSA}=\frac{12\mathrm{AM}R_{\text{field}}+12\mathrm{SMR}}{24\mathrm{SMR}}.$$

We repeated this for each iteration and then summarized FSA per species per size. We assumed that fish rested for 12 h (i.e., sleeping) (Marshall, 1972). As such, for all studied species, we assumed that they are active during the day and inactive during the night.

### 2.8 Assemblage‐level estimates

In 2016, reef fish communities were assessed across 13 sites on the outer fringing reef around Mo′orea using underwater visual censuses. During each census, a single diver swam along a 25 m transect and counted all fishes within a 2 m wide band. All fishes were identified to the species level and their total length was estimated to be the nearest 1 cm. Each transect covered an area of 50 m^2^, except Tiahura and Haapiti, which covered an area of 100 m^2^ each. At each site, three transects were performed, except for Tiahura and Haapiti where four and two transects were performed, respectively. We extracted data for our model species from this database, which resulted in 802 individuals across the six species. Then, we quantified the SMR and $\mathrm{AM}R_{\text{field}}$ for each individual using the above‐mentioned methodology. Finally, we calculated the total SMR and $\mathrm{AM}R_{\text{field}}$ of the fish assemblage composed of the six species at each site by summing across individual estimates.

## 3 RESULTS

### 3.1 Standard and maximum metabolic rates

**FIGURE 2:**
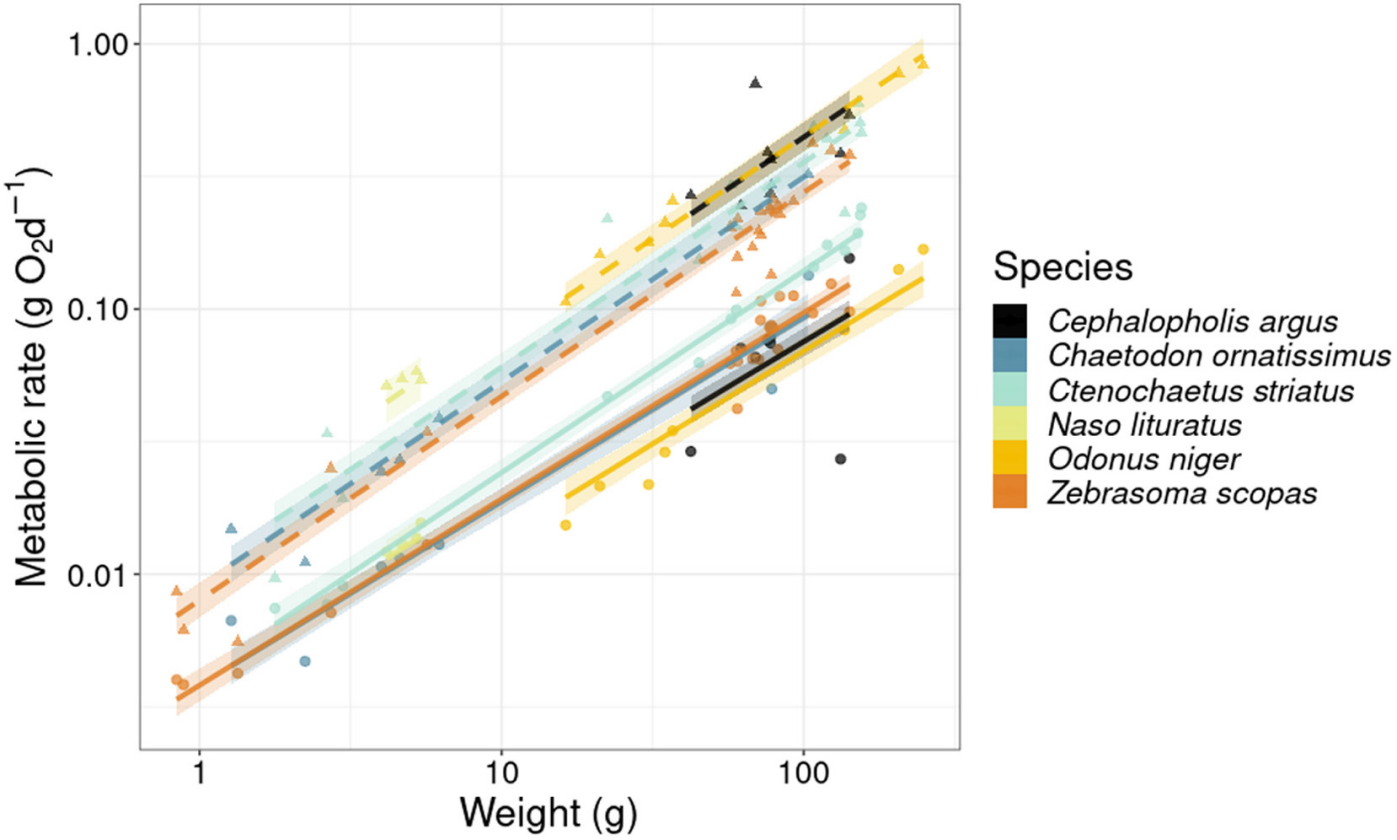
Linear regressions between log_10_‐transformed metabolic rate (g O_2_ d^−1^) and weight (g) for the study species. Symbols represent empirical measurements. Solid and dashed lines represent predicted mean standard metabolic rate (SMR) and maximum metabolic rate (MMR) values, respectively. Transparent areas are the 95% credible intervals of the fitted values of the regression

The regression model predicting MRs (log_10_ of SMR and MMR) as a function of log_10_ of body mass with varying slopes and intercepts per species had a Bayesian *R* ^2^ of .96 (Table 1; Figure 2). The average metabolic scaling exponent across species was 0.73 for SMR and 0.78 for MMR (Table 1). The median species‐specific scaling exponents varied between 0.68 and 0.76 for SMR and between 0.77 and 0.78 for MMR.

**TABLE 1:** Overview of species‐specific slope coefficients (scaling exponents) of the regression of $\mathrm{lo}g_{10}$‐transformed SMR and MMR on the function of $\mathrm{lo}g_{10}$‐transformed body mass

Species	SMR slope	SMR (mass = 1 g)	MMR slope	MMR (mass = 1 g)
*Cephalopholis argus*	0.68 (0.57; 0.77)	0.0033 (0.0019; 0.0047)	0.77 (0.69; 0.87)	0.0124 (0.0079; 0.0178)
*Chaetodon ornatissimus*	0.7 (0.6; 0.78)	0.0038 (0.0029; 0.0047)	0.77 (0.7; 0.85)	0.0091 (0.0069; 0.0117)
*Ctenochaetus striatus*	0.76 (0.68; 0.83)	0.0042 (0.0031; 0.0056)	0.77 (0.71; 0.84)	0.0103 (0.0078; 0.0137)
*Naso lituratus*	0.73 (0.57; 0.89)	0.0041 (0.0029; 0.0054)	0.78 (0.68; 0.93)	0.0146 (0.01; 0.0202)
*Odonus niger*	0.7 (0.58; 0.81)	0.0028 (0.0016; 0.0042)	0.77 (0.68; 0.85)	0.0129 (0.0081; 0.018)
*Zebrasoma scopas*	0.7 (0.64; 0.76)	0.0038 (0.003; 0.0046)	0.77 (0.72; 0.83)	0.008 (0.0063; 0.01)

*Note:* The intercept for each species is expressed as the back‐transformed value for an individual of 1 g. Values in between brackets represent the 95% CI.

### 3.2 Swimming speed

**FIGURE 3:**
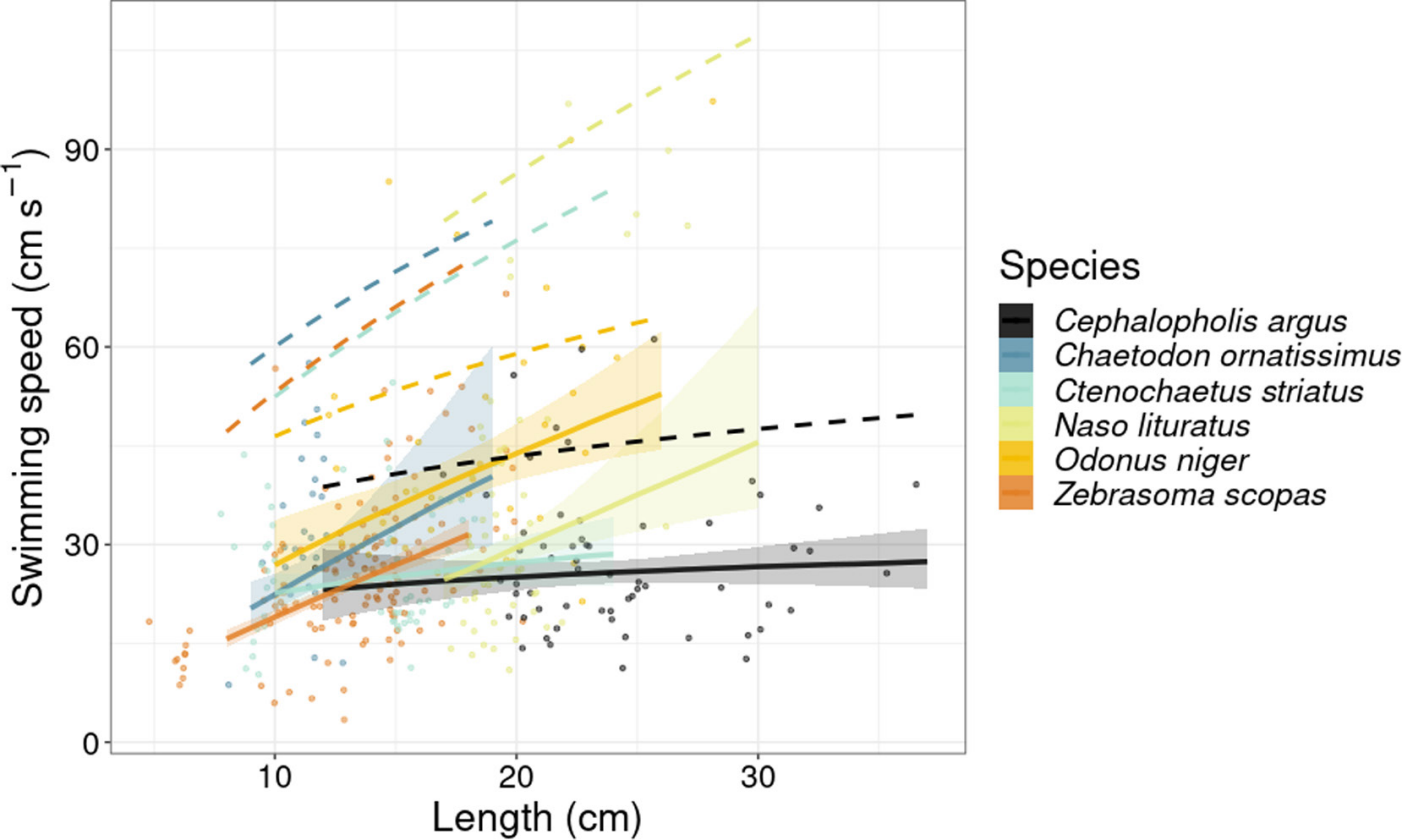
Linear regressions between log_10_‐transformed speed (cm s^−1^) and length (cm) for the six study species. Symbols represent the raw data of individuals measured through stereo‐video analysis. Solid lines and shaded areas represent the predicted mean back‐transformed values, and associated 95% credible interval of swimming speeds. The dashed lines represent the predicted maximum swimming speeds

The regression model predicting species‐specific swimming speed as a function of body size had a median Bayesian *R* ^2^ of .57 and its residual variance (σ) was 0.37. The average species‐specific slope values varied between 0.18 and 0.97 (Figure 3, Table S2). At the individual scale, the 95% credible interval of swimming speed predictions varied between 28.5 and 32.4 cm s^−1^ across all species and size classes. For maximum swimming speed, our model showed an increase in body size and aspect ratio (Table S3), with a median Bayesian *R* ^2^ of .46. We then used this model to estimate maximum swimming speeds (Figure 3).

### 3.3 FMR, FAS, and FSA estimations

**FIGURE 4:**
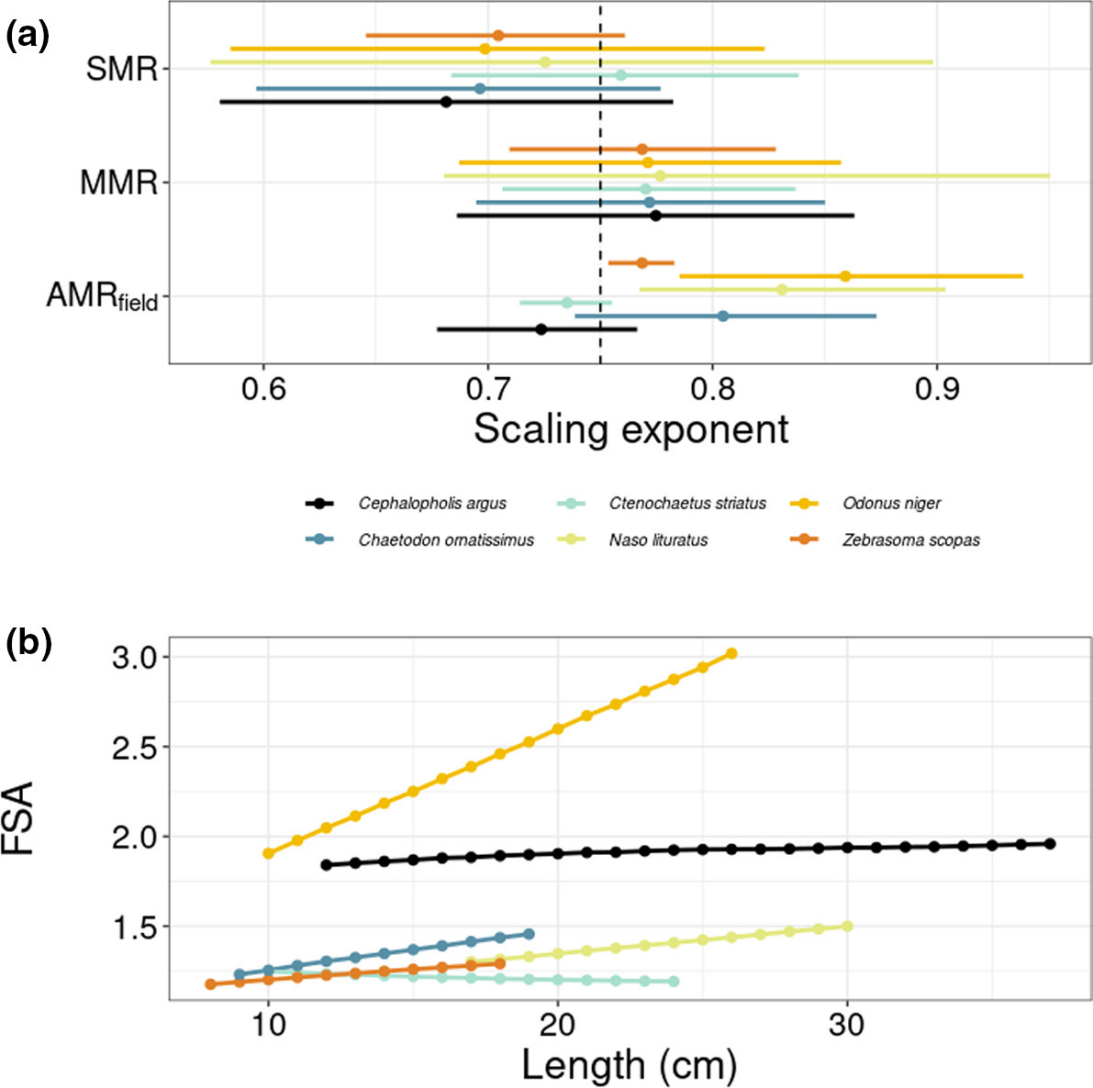
(a) Fitted scaling exponents for standard metabolic rate (SMR), maximum metabolic rate (MMR), and field metabolic rate (AMR_field_) based on slopes of the log_10_–log_10_ relationships between the metabolic rates (g O_2_ d^−1^) and body mass (g). Lines represent the 95% credible interval and dots indicate the average values. (b) Predicted average factorial scope for activity (FSA) for the six reef fish species across their body size range

We estimated $\mathrm{AM}R_{\text{field}}$, FAS, and FSA across the size range of our study species as observed in the monitoring dataset from Mo′orea in 2016. Across all species and size classes, the average $\mathrm{AM}R_{\text{field}}$ estimates ranged between 0.001 and 1.013 g O_2_ d^−1^ at the individual level (Table S4). FAS and FSA estimate range between 2.4 and 7.0, and between 1.2 and 3.2, respectively, across species and sizes. The scaling exponent of $\mathrm{AM}R_{\text{field}}$ was higher than the SMR exponent for all species, except for *C. striatus* (Figure 4a), hence, FSA increased with size for all those species (Figure 4b). The scaling exponent of $\mathrm{AM}R_{\text{field}}$ was considerably higher than the MMR exponents for *N. lituratus* and *O. niger*.

### 3.4 Assemblage‐level predictions

**FIGURE 5:**
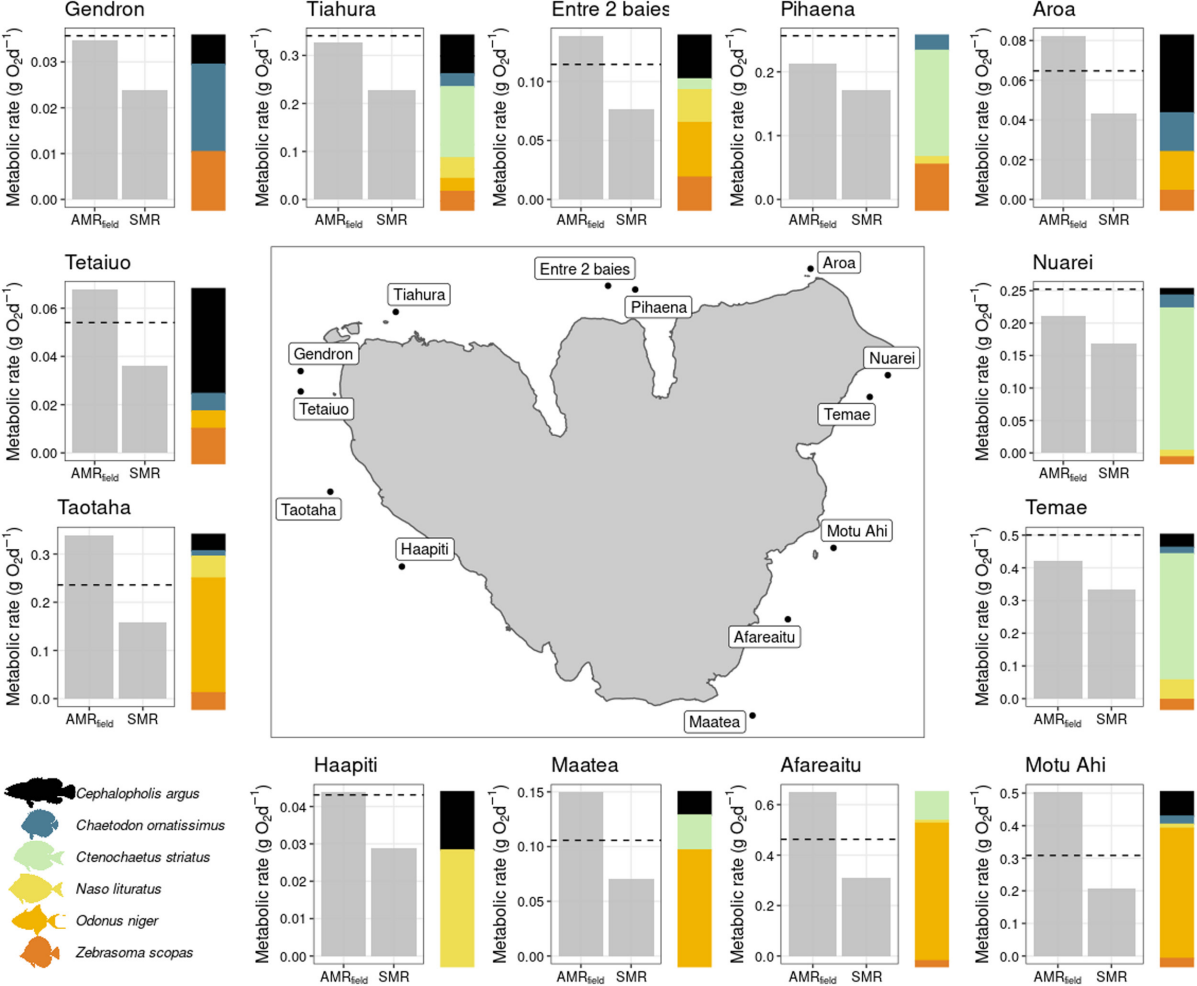
Field (AMR_field_) and standard metabolic rates (SMR) of an assemblage of six reef fish species at 13 sites around Mo′Orea, French Polynesia. Dashed lines represent 1.5 times the SMR as a reference. Colored bars display the relative abundances of the reef fish species at each site

Scaling up SMR and $\mathrm{AM}R_{\text{field}}$ to the assemblage level revealed major variation in the two estimates of metabolism, with average SMR (±SD) for this assemblage of six fish species across sites (ranging between 0.026 ± 0.009 and 0.325 ± 0.021 g O_2_ m^−2^ d^−1^; Figure 5) tending to be about half total $\mathrm{AM}R_{\text{field}}$ (ranging betwen 0.036 ± 0.014 and 0.465 ± 0.07 g O_2_ m^−2^ d^−1^). Spatial variation in total SMR and $\mathrm{AM}R_{\text{field}}$ reflected patterns in the relative abundance of the six study species across sites (Figures 5 and S4). Afareaitu, Maatea, Motu Ahi, Taotaha, and Tetaiuo, sites where *C. argus* and *O. niger* dominated the reef fish assemblage had a total $\mathrm{AM}R_{\text{field}}$ about twice as high as total SMR. On the contrary, sites dominated by *C. striatus* (50–95% of the total reef fish abundance) had a total $\mathrm{AM}R_{\text{field}}$ 1.27–1.41 times higher than total SMR (i.e., Nuarei, Pihaena, Temae, and Tiahura).

## 4 DISCUSSION

FMR is an essential organismal process that mediates consumption rates across the food web, thus influencing system‐wide fluxes of energy and nutrients. By coupling laboratory data on MRs with field observations of body size and swimming activity through stereo‐video analysis, we estimated the activity component of FMR (the $\mathrm{AM}R_{\text{field}}$). Further, we demonstrate that the FSA of reef fish species varies substantially across species, and that the metabolic scaling exponent of $\mathrm{AM}R_{\text{field}}$ can substantially exceed the canonical value of 0.75, which also affects community‐level estimates of MR. Therefore, our results highlight the potential pitfalls of estimating the community‐level MR of heterogeneous reef fish assemblages based on scaled‐up estimates of SMR instead of $\mathrm{AM}R_{\text{field}}$. We suggest that the coupling of physiological traits with stereo‐video analyses provides an opportunity to estimate FMRs of fishes in marine environments that allow for visual assessments.

The FSA can be an important parameter to predict the energy consumption of fishes in the wild (e.g., Schiettekatte et al., 2020). Our estimates of FSA were comparable to previous estimates for a small freshwater fish, in which the FSA was obtained through a combination of bioenergetic modeling and behavioral observations (~1.9; Trudel & Boisclair, 1996). In contrast, several other fish species may have a much higher $\mathrm{AM}R_{\text{field}}$ as locomotion has been reported to increase MR up to fivefold, and up to ninefold in tuna (*Thunnus albacares*) (Brill & Bushnell, 1991; Chabot et al., 2016). However, it is still challenging to quantify where $\mathrm{AM}R_{\text{field}}$ lies for most species.

The varying estimates of FSA may relate to the swimming speed and the aerobic capacity of the studied species (Clark et al., 2013; Killen et al., 2016). In our case study, the two fishes with the highest FSA were *O. niger* and *C. argus*, which appear to exploit about 45% and 60% of their aerobic scope in their natural environment, respectively. Therefore, *C. argus* has a high FSA mostly due to its high aerobic scope, while *O. niger* has the highest FSA in our case study both because of a high aerobic capacity and because it uses a larger proportion of it for swimming. On the other hand, fishes with a lower FSA (i.e., *C. ornatissimus*, *C. striatus*, and *Z. scopas*) were quite active, relative to their maximum swimming capacities, and exploited more than 50% of their aerobic scope. However, because their aerobic scope is low, so is their FSA.

These results corroborate the notion that $\mathrm{AM}R_{\text{field}}$ in fishes is strongly influenced by ecological traits, such as size, trophic level, and habitat use (Brown et al., 2004; Killen et al., 2016; Nash et al., 2015). Larger fishes tend to have a higher aerobic capacity than smaller species (Brown et al., 2004; Killen et al., 2007), and larger sizes in fishes permit the establishment of larger home ranges (Nash et al., 2015). Furthermore, predators often have a higher metabolic capacity, compared to herbivores, and pelagic fishes often have higher metabolic potential than benthic fishes, as they have high locomotory demands because of their mobility in a 3D environment (Killen et al., 2016; Nash et al., 2015). Pairwise comparisons among our study species (e.g., the herbivorous *Z. scopas* vs. the carnivorous *C. argus* or the benthopelagic *C. striatus* vs. the epipelagic *O. niger*) strongly support an ecological basis for metabolic differentiation.

Beyond interspecific differences, our results suggest that $\mathrm{AM}R_{\text{field}}$ scales differently with body mass compared to SMR or MMR. The SMRs of our study species varied predictably with body mass, in accordance with the metabolic theory of ecology (Brown et al., 2004), with the average slope value approximating the allometric scaling exponent of 0.75 often found empirically and predicted theoretically by West et al. (1997). In contrast, half of the species (i.e., *Z. scopas*, *N. lituratus*, and *O. niger*) had a scaling exponent for $\mathrm{AM}R_{\text{field}}$, that exceeded 0.75 with 95% credibility. In particular, the FSA for *N. lituratus*, and *O. niger* was positively correlated with body size, suggesting that large individuals consume more oxygen in their natural environment than previously assumed. For other species, such as *C. argus* and *C. striatus*, the scaling exponent of $\mathrm{AM}R_{\text{field}}$ was similar to that of SMR implying a negligible effect of activity on metabolic scaling. Importantly, there appears to be a higher interspecific variability of the scaling exponent for $\mathrm{AM}R_{\text{field}}$ compared to that for SMR and MMR. This underlines the importance of both species identity and body size when estimating FMR.

Scaling up, community‐level SMRs should vary predictively with both community composition and intraspecific size structure (Allen et al., 2005; Barneche et al., 2014). However, failing to account for the increased variation in scaling exponents of FMR may lead to severe underestimates of the contribution of large mobile fishes to the total respiration of fish communities. Indeed, comparing our assemblage‐level estimates based on SMR with assemblage‐level estimates based on $\mathrm{AM}R_{\text{field}}$ reveals the potential pitfalls of using SMR to study community‐level MRs (e.g., Cheung et al., 2013; Deutsch et al., 2015; Holt & Jørgensen, 2015). The ratio between community‐level $\mathrm{AM}R_{\text{field}}$ and SMR is highly variable, thus suggesting that universal corrections to convert laboratory‐estimated SMR into $\mathrm{AM}R_{\text{field}}$ are likely unreliable. For example, communities with a similar biomass and size structure may be considered as having a similar MR when using SMR as a proxy. However, if a community includes species that have a much higher metabolic scaling exponent, the role of large individuals, and thus the community‐level MR may be underestimated severely. Thus, it would be important to consider a higher variation in metabolic scaling of FMR than previously assumed if we want to estimate energy flow in fish communities.

While our approach offers a novel way to estimate the activity rate and MR of fishes, it comes with limitations. First, we extrapolated maximum swimming speeds for our study species based on literature data to reconstruct the relationship between MR and swimming speed (Fulton, 2007). Although we accounted for size, family, variation in body shapes, and a proxy for swimming ability, swimming speed across species within a family and body shape may still differ substantially, introducing potential bias to our calculations. Further, our method relies on the assumption that MR varies predictably with swimming speed following a traditional power function (Brett, 1964; Korsmeyer et al., 2002). While this power function has been found to accurately predict MR for multiple species, more complex functions may be optimal for others, for example incorporating a plateau at the highest swimming speeds (Roche et al., 2013). Finally, the method we used to define MMR (i.e., the chase method) could have introduced additional bias. MMR does not differ between post‐exercise (e.g., chase) and sustained swimming across multiple species (Killen et al., 2017). However, there is alternative evidence from coral reef fishes suggesting that they can in fact achieve a higher maximum rate of oxygen uptake while swimming compared to after a chase (Roche et al., 2013; Rummer et al., 2016). Future studies could resolve all of the above‐mentioned issues by measuring swimming speed and respiration rate simultaneously in the laboratory and our approach can easily be adapted when additional data become available.

Furthermore, we quantified FSA assuming that fishes' spontaneous swimming activity follows strict circadian cycles, with all activity occurring diurnally. However, the activity patterns of reef fishes are often flexible (Zhdanova & Reebs, 2006). While, in principle, all our studied families are diurnally active, some species (e.g., Serranidae) can be nocturnally active (Mourier et al., 2016). Thus, our assumption can cause potential underestimates of FSA in *C. argus* and other species with more flexible circadian activity patterns. Currently, stereo‐video recordings are unable to quantify fish swimming speeds at night, as measurements are inaccurate and imprecise in darkness and poor visibility (Neuswanger et al., 2016). Infrared lighting in stereo‐video recordings could provide an opportunity to observe nocturnal behavior and movement in fishes, but only observations in close proximity are likely to be fruitful because of the limited range of infrared light (Bassett & Montgomery, 2011).

Finally, while $\mathrm{AM}R_{\text{field}}$ represents an improved estimate of energy expenditure in the field, it still lacks components such as reproduction and digestion. Digestion (often expressed as specific dynamic action [SDA]) can be a large component of the energy budget of certain fishes (e.g., ~17% of SMR; Holt & Jørgensen, 2015). SDA can be measured in the laboratory, where a fish is given a meal and the resulting increase in oxygen consumption is measured for the duration of the digestion of this meal. SDA relates predictably to both the meal size and body mass of a fish (Secor, 2009), but using this relationship to calculate the SDA of species in natural communities is not feasible. It is nearly impossible to track the frequency of meals and meal sizes of fishes in the wild, even though some bioenergetic modeling allows for an approximation of daily consumption rates (e.g., Schiettekatte et al., 2020). Further, these experiments are largely based on predatory fishes, and do not necessarily represent natural feeding behavior as many fishes do not consume and digest a meal fully before eating the next meal. Notably, herbivores, detritivores, and planktivores, feed constantly, and their energy expenditure related to digestion is understudied. Therefore, we stress the need for more research on the energy consumption of digestion across a wide range of fishes to achieve improved FMR approximations for fish communities in the wild.

Despite these limitations, our proposed method may help us understand some of the variations in $\mathrm{AM}R_{\text{field}}$ among reef fishes, which is necessary to understand ecosystem‐level estimates of elemental fluxes. So far, the quantification of $\mathrm{AM}R_{\text{field}}$ is limited to laboratory techniques that are reliant on destructive sampling (analysis of trace elements in otoliths; Chung et al., 2019), or restricted to species that are big enough to be tagged with biotelemetry equipment (Brodie et al., 2016; Treberg et al., 2016). When combined with respirometry trials, stereo‐video offers a non‐destructive and non‐invasive alternative to these techniques that can be applied to all species that can be reliably observed using in situ cameras. While the post‐hoc treatment of the stereo‐video outputs demands significant time and effort, the development of open‐source software to automate data collection from the video will greatly strengthen our ability and non‐destructive approach to quantifying reef fish $\mathrm{AM}R_{\text{field}}$ (Bassett & Montgomery, 2011; Guénard et al., 2008).

## AUTHOR CONTRIBUTIONS

**Nina Monique Dominique Schiettekatte:** Conceptualization (lead); data curation (equal); formal analysis (equal); investigation (equal); methodology (equal); project administration (equal); resources (equal); software (equal); supervision (equal); visualization (equal); writing – original draft (equal); writing – review and editing (lead). **Francesca Conte:** Data curation (equal); formal analysis (equal); investigation (equal); methodology (equal); resources (equal); software (equal); visualization (equal); writing – original draft (equal). **Beverly French:** Methodology (supporting); validation (supporting); writing – review and editing (supporting). **Simon J. Brandl:** Funding acquisition (equal); investigation (supporting); supervision (supporting); validation (equal); visualization (supporting); writing – review and editing (equal). **Christopher Fulton:** Investigation (supporting); writing – review and editing (supporting). **Alexandre Mercière:** Data curation (equal); methodology (equal); project administration (equal). **Tommy Norin:** Data curation (equal); methodology (equal); validation (equal); writing – review and editing (equal). **Sebastian Villéger:** Supervision (equal); validation (equal); writing – review and editing (equal). **Valerianio Parravicini:** Conceptualization (supporting); funding acquisition (lead); investigation (equal); supervision (lead); validation (equal); writing – review and editing (equal).

### OPEN RESEARCH BADGES

This article has earned an Open Data badge for making publicly available the digitally‐shareable data necessary to reproduce the reported results. The data is available at https://github.com/nschiett/activity_rate_fishes.

## Supporting information


Appendix S1

## Data Availability

All data and code needed to reproduce results, tables, and figures are provided at https://github.com/nschiett/activity_rate_fishes.

## References

[ece39084-bib-0001] Allen, A. P. , Gillooly, J. F. , & Brown, J. H. (2005). Linking the global carbon cycle to individual metabolism. Functional Ecology, 19(2), 202–213. 10.1111/j.1365-2435.2005.00952.x

[ece39084-bib-0002] Allgeier, J. E. , Layman, C. A. , Mumby, P. J. , & Rosemond, A. D. (2014). Consistent nutrient storage and supply mediated by diverse fish communities in coral reef ecosystems. Global Change Biology, 20(8), 2459–2472. 10.1111/gcb.12566 24692262

[ece39084-bib-0003] Barneche, D. R. , Kulbicki, M. , Floeter, S. R. , Friedlander, A. M. , Maina, J. , & Allen, A. P. (2014). Scaling metabolism from individuals to reef‐fish communities at broad spatial scales. Ecology Letters, 17(9), 1067–1076. 10.1111/ele.12309 24943721

[ece39084-bib-0004] Bassett, D. K. , & Montgomery, J. C. (2011). Investigating nocturnal fish populations in situ using baited underwater video: With special reference to their olfactory capabilities. Journal of Experimental Marine Biology and Ecology, 409(1–2), 194–199. 10.1016/j.jembe.2011.08.019

[ece39084-bib-0005] Binning, S. A. , Roche, D. G. , & Layton, C. (2013). Ectoparasites increase swimming costs in a coral reef fish. Biology Letters, 9(1), 20120927. 10.1098/rsbl.2012.0927 23193046 PMC3565510

[ece39084-bib-0006] Bokma, F. (2004). Evidence against universal metabolic allometry. Functional Ecology, 18, 184–187.

[ece39084-bib-0007] Bozec, Y.‐M. , Gascuel, D. , & Kulbicki, M. (2004). Trophic model of lagoonal communities in a large open atoll (uvea, loyalty islands, New Caledonia). Aquatic Living Resources, 17(2), 151–162. 10.1051/alr:2004024

[ece39084-bib-0008] Brandl, S. J. , Rasher, D. B. , Côté, I. M. , Casey, J. M. , Darling, E. S. , Lefcheck, J. S. , & Duffy, J. E. (2019). Coral reef ecosystem functioning: Eight core processes and the role of biodiversity. Frontiers in Ecology and the Environment, 17, 445–454. 10.1002/fee.2088

[ece39084-bib-0009] Brandl, S. J. , Tornabene, L. , Goatley, C. H. R. , Casey, J. M. , Morais, R. A. , Côté, I. M. , Baldwin, C. C. , Parravicini, V. , Schiettekatte, N. M. D. , & Bellwood, D. R. (2019). Demographic dynamics of the smallest marine vertebrates fuel coral reef ecosystem functioning. Science, 364(6446), 1189–1192. 10.1126/science.aav3384 31123105

[ece39084-bib-0010] Brett, J. R. (1964). The respiratory metabolism and swimming performance of young sockeye Salmon. Journal of the Fisheries Research Board of Canada, 21(5), 1183–1226. 10.1139/f64-103

[ece39084-bib-0011] Brill, R. W. , & Bushnell, P. G. (1991). Metabolic and cardiac scope of high energy demand teleosts, the tunas. Canadian Journal of Zoology, 69(7), 2002–2009. 10.1139/z91-279

[ece39084-bib-0012] Brodie, S. , Taylor, M. D. , Smith, J. A. , Suthers, I. M. , Gray, C. A. , & Payne, N. L. (2016). Improving consumption rate estimates by incorporating wild activity into a bioenergetics model. Ecology and Evolution, 6(8), 2262–2274. 10.1002/ece3.2027 27069576 PMC4782250

[ece39084-bib-0013] Brown, J. H. , Gillooly, J. F. , Allen, A. P. , Savage, V. M. , & West, G. B. (2004). Toward a metabolic theory of ecology. Ecology, 85(7), 1771–1789. 10.1890/03-9000

[ece39084-bib-0014] Burkner, P. C. (2017). Brms: An R package for Bayesian multilevel models using Stan. Journal of Statistical Software, 80, 1–28. 10.18637/jss.v080.i01

[ece39084-bib-0015] Butail, S. , & Paley, D. A. (2012). Three‐dimensional reconstruction of the fast‐start swimming kinematics of densely schooling fish. Journal of the Royal Society Interface, 9(66), 77–88. 21642367 10.1098/rsif.2011.0113 PMC3223621

[ece39084-bib-0016] Cardinale, B. J. , Duffy, J. E. , Gonzalez, A. , Hooper, D. U. , Perrings, C. , Venail, P. , Narwani, A. , Mace, G. M. , Tilman, D. , Wardle, D. A. , Kinzig, A. P. , Daily, G. C. , Loreau, M. , Grace, J. B. , Larigauderie, A. , Srivastava, D. S. , & Naeem, S. (2012). Biodiversity loss and its impact on humanity. Nature, 486, 59–67. 10.1038/nature11148 22678280

[ece39084-bib-0017] Chabot, D. , Steffensen, J. F. , & Farrell, A. P. (2016). The determination of standard metabolic rate in fishes. Journal of Fish Biology, 88(1), 81–121. 10.1111/jfb.12845 26768973

[ece39084-bib-0018] Cheung, W. W. L. , Sarmiento, J. L. , Dunne, J. , Frölicher, T. L. , Lam, V. W. Y. , Palomares, M. L. D. , Watson, R. , & Pauly, D. (2013). Shrinking of fishes exacerbates impacts of global ocean changes on marine ecosystems. Nature Climate Change, 3(3), 254–258. 10.1038/nclimate1691

[ece39084-bib-0019] Chung, M.‐T. , Trueman, C. N. , Godiksen, J. A. , Holmstrup, M. E. , & Grønkjær, P. (2019). Field metabolic rates of teleost fishes are recorded in otolith carbonate. Communications Biology, 2(1), 1–10. 10.1038/s42003-018-0266-5 30675522 PMC6338665

[ece39084-bib-0021] Clark, T. D. , Sandblom, E. , & Jutfelt, F. (2013). Aerobic scope measurements of fishes in an era of climate change: Respirometry, relevance and recommendations. Journal of Experimental Biology, 216, 2771–2782. 10.1242/jeb.084251 23842625

[ece39084-bib-0022] Clarke, A. , & Johnston, N. M. (1999). Scaling of metabolic rate with body mass and temperature in teleost fish. Jornal of Animal Ecology, 68, 893–905.

[ece39084-bib-0023] Cruz‐Font, L. , Shuter, B. J. , & Blanchfield, P. J. (2016). Energetic costs of activity in wild lake trout: a calibration study using acceleration transmitters and positional telemetry. Canadian Journal of Fisheries and Aquatic Sciences, 73(8), 1237–1250. 10.1139/cjfas-2015-0323

[ece39084-bib-0024] Deutsch, C. , Ferrel, A. , Seibel, B. , Pörtner, H. O. , & Huey, R. B. (2015). Climate change tightens a metabolic constraint on marine habitats. Science, 348(6239), 1132–1135. 10.1126/science.aaa1605 26045435

[ece39084-bib-0025] Froese, R. , & Pauly, D. (2018). FishBase. World Wide Web Electronic Publication.

[ece39084-bib-0026] Froese, R. , Thorson, J. T. , & Reyes, R. B. (2014). A Bayesian approach for estimating length‐weight relationships in fishes. Journal of Applied Ichthyology, 30(1), 78–85. 10.1111/jai.12299

[ece39084-bib-0027] Fry, F. (1947). Effects of the environment on animal activity. University of Toronto Studies Biological Series, No. 55 (pp. 1–62). Ontario Fisheries Research Laboratory Publication.

[ece39084-bib-0028] Fry, F. E. J. (1957). The aquatic respiration of fish. In M. E. Brown (Ed.), The physiology of fishes (pp. 1–63). Elsevier. 10.1016/b978-1-4832-2817-4.50006-8

[ece39084-bib-0029] Fulton, C. J. (2007). Swimming speed performance in coral reef fishes: Field validations reveal distinct functional groups. Coral Reefs, 26(2), 217–228. 10.1007/s00338-007-0195-0

[ece39084-bib-0030] Gillooly, J. F. , Brown, J. H. , West, G. B. , Savage, V. M. , & Charnov, E. L. (2001). Effects of size and temperature on metabolic rate. Science, 293(5538), 2248–2251. 10.1126/science.1061967 11567137

[ece39084-bib-0031] Glazier, D. S. (2005). Beyond the ‘3/4‐power law’: Variation in the intra‐ and interspecific scaling of metabolic rate in animals. Biological Reviews of the Cambridge Philosophical Society, 80(4), 611–662. 10.1017/S1464793105006834 16221332

[ece39084-bib-0032] Gräns, A. , Axelsson, M. , Pitsillides, K. , Olsson, C. , Höjesjö, J. , Kaufman, R. C. , & Cech, J. J. (2009). A fully implantable multi‐channel biotelemetry system for measurement of blood flow and temperature: A first evaluation in the green sturgeon. Hydrobiologia, 619(1), 11–25. 10.1007/s10750-008-9578-7

[ece39084-bib-0033] Guénard, G. , Boisclair, D. , Ugedal, O. , Forseth, T. , & Jonsson, B. (2008). Comparison between activity estimates obtained using bioenergetic and behavioural analyses. Canadian Journal of Fisheries and Aquatic Sciences, 65(8), 1705–1720. 10.1139/F08-080

[ece39084-bib-0034] Halpern, B. S. , Walbridge, S. , Selkoe, K. A. , Kappel, C. V. , Micheli, F. , D'Agrosa, C. , Casey, K. S. , Ebert, C. , Fox, H. E. , Fujita, R. , Heinemann, D. , Lenihan, H. S. , Madin, E. M. , Perry, M. T. , Selig, E. R. , Spalding, M. , Steneck, R. , & Watson, R. (2008). A global map of human impact on marine ecosystems. Science, 319(5865), 948–952. 10.1126/science.1149345 18276889

[ece39084-bib-0035] Halsey, L. G. , Killen, S. S. , Clark, T. D. , & Norin, T. (2018). Exploring key issues of aerobic scope interpretation in ectotherms: Absolute versus factorial. Reviews in Fish Biology and Fisheries, 18, 405–415. 10.1007/s11160-018-9516-3

[ece39084-bib-0036] Holt, R. E. , & Jørgensen, C. (2015). Climate change in fish: Effects of respiratory constraints on optimal life history and behaviour. Biology Letters, 11(2), 2014–1032. 10.1098/rsbl.2014.1032 PMC4360111 25673000

[ece39084-bib-0037] Hughes, N. F. , & Kelly, L. H. (1996). New techniques for 3‐D video tracking of fish swimming movements in still or flowing water. Canadian Journal of Fisheries and Aquatic Sciences, 53(11), 2473–2483. 10.1139/f96-200

[ece39084-bib-0038] Killen, S. S. , Costa, I. , Brown, J. A. , & Gamperl, A. K. (2007). Little left in the tank: Metabolic scaling in marine teleosts and its implications for aerobic scope. Proceedings of the Royal Society B: Biological Sciences, 274(1608), 431–438. 10.1098/rspb.2006.3741 PMC1702384 17164208

[ece39084-bib-0039] Killen, S. S. , Glazier, D. S. , Rezende, E. L. , Clark, T. D. , Atkinson, D. , Willener, A. S. T. , & Halsey, L. G. (2016). Ecological influences and morphological correlates of resting and maximal metabolic rates across teleost fish species. The American Naturalist, 187(5), 592–606. 10.1086/685893 27104992

[ece39084-bib-0040] Killen, S. S. , Norin, T. , & Halsey, L. G. (2017). Do method and species lifestyle affect measures of maximum metabolic rate in fishes? Journal of Fish Biology, 90, 1037–1046. 10.1111/jfb.13195 27778342 PMC5347950

[ece39084-bib-0041] Korsmeyer, K. E. , Steffensen, J. F. , & Herskin, J. (2002). Energetics of median and paired fin swimming, body and caudal fin swimming, and gait transition in parrotfish (Scarus schlegeli) and triggerfish (Rhinecanthus aculeatus). The Journal of Experimental Biology, 205(Pt 9), 1253–1263. 11948202 10.1242/jeb.205.9.1253

[ece39084-bib-0042] Lucas, M. C. , Johnstone, A. D. F. , & Priede, I. G. (1993). Use of physiological telemetry as a method of estimating metabolism of fish in the natural environment. Transactions of the American Fisheries Society, 122(5), 822–833. 10.1577/1548-8659(1993)122<0822:UOPTAA>2.3.CO;2

[ece39084-bib-0043] Marshall, D. J. , & White, C. R. (2019). Have we outgrown the existing models of growth? Elsevier Ltd. 10.1016/j.tree.2018.10.005 30396685

[ece39084-bib-0044] Marshall, N. B. (1972). Normal sleep in animals and man [abridged]: Sleep in fishes [abstract]. Journal of the Royal Society of Medicine, 16, 177. 10.1177/003591577206500235

[ece39084-bib-0045] Morais, R. A. , & Bellwood, D. R. (2019). Pelagic subsidies underpin fish productivity on a degraded coral reef. Current Biology, 29(9), 1521–1527.e6. 10.1016/j.cub.2019.03.044 31006572

[ece39084-bib-0046] Motulsky, H. J. , & Brown, R. E. (2006). Detecting outliers when fitting data with nonlinear regression: A new method based on robust nonlinear regression and the false discovery rate. BMC Bioinformatics, 7, 1–20. 10.1186/1471-2105-7-123 16526949 PMC1472692

[ece39084-bib-0047] Mourier, J. , Maynard, J. , Parravicini, V. , Ballesta, L. , Clua, E. , Domeier, M. L. , & Planes, S. (2016). Extreme inverted trophic pyramid of reef sharks supported by spawning groupers. Current Biology, 26(15), 2011–2016. 10.1016/j.cub.2016.05.058 27476598

[ece39084-bib-0048] Murchie, K. J. , Cooke, S. J. , Danylchuk, A. J. , & Suski, C. D. (2011). Estimates of field activity and metabolic rates of bonefish (Albula vulpes) in coastal marine habitats using acoustic tri‐axial accelerometer transmitters and intermittent‐flow respirometry. Journal of Experimental Marine Biology and Ecology, 396(2), 147–155. 10.1016/j.jembe.2010.10.019

[ece39084-bib-0049] Nagy, K. A. (2005). Field metabolic rate and body size. The Company of Biologists Ltd. 10.1242/jeb.01553 15855393

[ece39084-bib-0050] Nash, K. L. , Welsh, J. Q. , Graham, N. A. J. , & Bellwood, D. R. (2015). Home‐range allometry in coral reef fishes: Comparison to other vertebrates, methodological issues and management implications. Oecologia, 177(1), 73–83. 10.1007/s00442-014-3152-y 25424157

[ece39084-bib-0051] Neuswanger, J. R. , Wipfli, M. S. , Rosenberger, A. E. , & Hughes, N. F. (2016). Measuring fish and their physical habitats: Versatile 2D and 3D video techniques with user‐friendly software. Canadian Journal of Fisheries and Aquatic Sciences, 73(12), 1861–1873. 10.1139/cjfas-2016-0010

[ece39084-bib-0052] Norin, T. , & Clark, T. D. (2016). Measurement and relevance of maximum metabolic rate in fishes. Journal of Fish Biology, 88(1), 122–151. 10.1111/jfb.12796 26586591

[ece39084-bib-0054] Reich, P. B. , Tilman, D. , Isbell, F. , Mueller, K. , Hobbie, S. E. , Flynn, D. F. B. , & Eisenhauer, N. (2012). Impacts of biodiversity loss escalate through time as redundancy fades. Science, 336(6081), 589–592. 10.1126/science.1217909 22556253

[ece39084-bib-0055] Roche, D. G. , Binning, S. A. , Bosiger, Y. , Johansen, J. L. , & Rummer, J. L. (2013). Finding the best estimates of metabolic rates in a coral reef fish. Journal of Experimental Biology, 216(11), 2103–2110. 10.1242/jeb.082925 23470659

[ece39084-bib-0056] Rummer, J. L. , Binning, S. A. , Roche, D. G. , & Johansen, J. L. (2016). Methods matter: Considering locomotory mode and respirometry technique when estimating metabolic rates of fishes. Conservation Physiology, 4(1), cow008. 10.1093/conphys/cow008 27382471 PMC4922262

[ece39084-bib-0057] Schiettekatte, N. M. D. , Barneche, D. R. , Villéger, S. , Allgeier, J. E. , Burkepile, D. E. , Brandl, S. J. , Casey, J. M. , Mercière, A. , Munsterman, K. S. , Morat, F. , & Parravicini, V. (2020). Nutrient limitation, bioenergetics and stoichiometry: A new model to predict elemental fluxes mediated by fishes. Functional Ecology, 34(9), 1857–1869. 10.1111/1365-2435.13618

[ece39084-bib-0058] Secor, S. M. (2009). Specific dynamic action: A review of the postprandial metabolic response. Journal of Comparative Physiology. B, Biochemical, Systemic, and Environmental Physiology, 179, 1–56. 10.1007/s00360-008-0283-7 18597096

[ece39084-bib-0059] Steffensen, J. F. (1989). Some errors in respirometry of aquatic breathers: How to avoid and correct for them. Fish Physiology and Biochemistry, 6(1), 49–59. 10.1007/BF02995809 24226899

[ece39084-bib-0060] Svendsen, M. B. S. , Bushnell, P. G. , & Steffensen, J. F. (2016). Design and setup of intermittent‐flow respirometry system for aquatic organisms. Journal of Fish Biology, 88(1), 26–50. 10.1111/jfb.12797 26603018

[ece39084-bib-0061] Tamayo, N. C. A. , Anticamara, J. A. , & Acosta‐Michlik, L. (2018). National Estimates of values of Philippine Reefs' ecosystem services. Ecological Economics, 146, 633–644. 10.1016/j.ecolecon.2017.12.005

[ece39084-bib-0062] Tilman, D. , Isbell, F. , & Cowles, J. M. (2014). Biodiversity and ecosystem functioning. Annual Review of Ecology, Evolution, and Systematics, 45(1), 471–493. 10.1146/annurev-ecolsys-120213-091917

[ece39084-bib-0063] Torres, J. J. , & Childress, J. J. (1983). Relationship of oxygen consumption to swimming speed in Euphausia pacifica ‐ 1. Effects of temperature and pressure. Marine Biology, 74(1), 79–86. 10.1007/BF00394278

[ece39084-bib-0064] Treberg, J. R. , Killen, S. S. , MacCormack, T. J. , Lamarre, S. G. , & Enders, E. C. (2016). Estimates of metabolic rate and major constituents of metabolic demand in fishes under field conditions: Methods, proxies, and new perspectives. Comparative Biochemistry and Physiology ‐ Part A: Molecular and Integrative Physiology, 202, 10–22. 10.1016/j.cbpa.2016.04.022 27139083

[ece39084-bib-0065] Trudel, M. , & Boisclair, D. (1996). Estimation of fish activity costs using underwater video cameras. Journal of Fish Biology, 48(1), 40–53. 10.1111/j.1095-8649.1996.tb01417.x

[ece39084-bib-0066] Venter, O. , Sanderson, E. W. , Magrach, A. , Allan, J. R. , Beher, J. , Jones, K. R. , Possingham, H. P. , Laurance, W. F. , Wood, P. , Fekete, B. M. , Levy, M. A. , & Watson, J. E. M. (2016). Sixteen years of change in the global terrestrial human footprint and implications for biodiversity conservation. Nature Communications, 7(1), 1–11. 10.1038/ncomms12558 PMC4996975 27552116

[ece39084-bib-0067] Villéger, S. , Brosse, S. , Mouchet, M. , Mouillot, D. , & Vanni, M. J. (2017). Functional ecology of fish: Current approaches and future challenges. Aquatic Sciences, 79(4), 783–801. 10.1007/s00027-017-0546-z

[ece39084-bib-0068] Vinberg, G. (1960). Rate of metabolism and food requirements of fishes. Fisheries Research Board of Canada Biological Station.

[ece39084-bib-0069] Von Bertalanffy, L. (1957). Quantitative laws in metabolism and growth. The Quarterly Review of Biology, 32(3), 217–231. 10.1086/401873 13485376

[ece39084-bib-0070] Webster, M. D. , & Weathers, W. W. (1989). Validation of single‐sample doubly labeled water method. American Journal of Physiology. Regulatory, Integrative and Comparative Physiology, 256(2), R572–R576. 10.1152/ajpregu.1989.256.2.R572 2492774

[ece39084-bib-0071] West, G. B. , Brown, J. H. , & Enquist, B. J. (1997). A general model for the origin of allometric scaling laws in biology. Science, 276(5309), 122–126. 10.1126/science.276.5309.122 9082983

[ece39084-bib-0072] Wilson, S. K. , Adjeroud, M. , Bellwood, D. R. , Berumen, M. L. , Booth, D. , Bozec, Y.‐M. , Chabanet, P. , Cheal, A. , Cinner, J. , Depczynski, M. , Feary, D. A. , Gagliano, M. , Graham, N. A. , Halford, A. R. , Halpern, B. S. , Harborne, A. R. , Hoey, A. S. , Holbrook, S. J. , Jones, G. P. , … Syms, C. (2010). Crucial knowledge gaps in current understanding of climate change impacts on coral reef fishes. Journal of Experimental Biology, 213(6), 894–900. 10.1242/jeb.037895 20190114

[ece39084-bib-0073] Zhdanova, I. V. , & Reebs, S. G. (2006). Circadian rhythms in fish. Fish Physiology, 24, 197–238. 10.1016/S1546-5098(05)24006-2

