# Supplementary material for: Combining stereo‐video monitoring and physiological trials to estimate reef fish metabolic demands in the wild
Source: Ecol Evol. 2022 Jul 5;12(7):e9084. doi: 10.1002/ece3.9084 (PMC9254678; doi:10.1002/ece3.9084)
Supplement: Supplementary file 1 — Appendix S1 [file ECE3-12-e9084-s001.docx]

## Supporting information


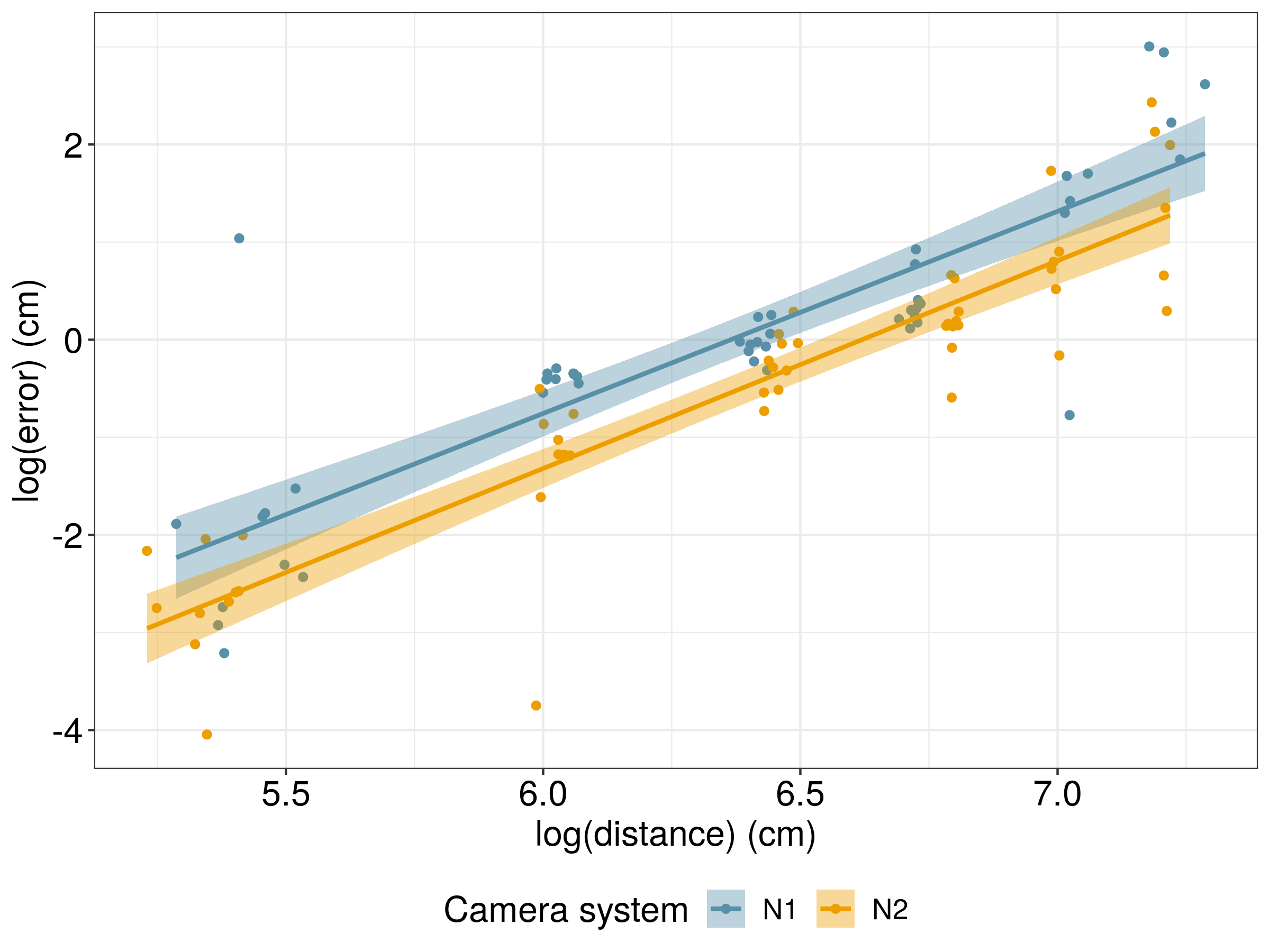


**Figure S1.** Linear regressions between the error (cm) in measurements collected by video analysis and the distance (cm) from the nearest camera for both underwater stereo-video systems. Each color represents an underwater stereo-camera system used in this study. Shaded areas show the linear regression standard errors.

**Table S2:** Overview of species-specific slope and intercept coefficients for the regression of log_10_-transformed swimming speed on log_10_-transformed body length (in cm). The 95% credible interval is displayed in the parentheses.

| Species | Slope log_10_(length) | Intercept |
| --- | --- | --- |
| *Cephalopholis argus* | 0.14 (-0.37;0.62) | 1.21 (0.53;1.88) |
| *Chaetodon ornatissimus* | 0.92 (0.05;2.08) | 0.43 (-0.79;1.36) |
| *Ctenochaetus striatus* | 0.26 (-0.14;0.73) | 1.09 (0.55;1.54) |
| *Naso lituratus* | 1.06 (0.28;2.28) | 0.09 (-1.48;1.11) |
| *Odonus niger* | 0.71 (0.11;1.22) | 0.72 (0.08;1.46) |
| *Zebrasoma scopas* | 0.86 (0.61;1.08) | 0.42 (0.16;0.69) |

**Table S3:** Overview of regression parameters of log_10_-transformed maximum swimming speed as function of log_10_-transformed body length (in cm), aspect ratio, and with varying intercepts and slopes per interaction of family and body shape.

| Family | Body shape | Slope log_10_(length) | Slope aspect ratio | Intercept |
| --- | --- | --- | --- | --- |
| Acanthuridae | fusiform/normal | 0.28 (-0.26;0.86) | 0.09 (0.05;0.14) | 1.2 (0.57;1.84) |
| Acanthuridae | short and/or deep | 0.53 (0.24;0.86) | 0.09 (0.05;0.14) | 1.01 (0.63;1.33) |
| Balistidae | short and/or deep | 0.32 (-0.19;0.91) | 0.09 (0.05;0.14) | 1.17 (0.5;1.76) |
| Chaetodontidae | short and/or deep | 0.43 (0.07;0.8) | 0.09 (0.05;0.14) | 1.13 (0.76;1.47) |
| Serranidae | fusiform/normal | 0.22 (-0.22;0.71) | 0.09 (0.05;0.14) | 1.22 (0.64;1.76) |

**Table S4:** Overview of average species- and size-specific estimates of standard metabolic rate (SMR, in g O_2_ d^−1^), maximum metabolic rate (MMR, in g O_2_ d^−1^), field active metabolic rate (AMR_field_, in g O_2_ d^−1^), factorial aerobic scope (FAS), and factorial scope for activity (FSA). Length is expressed in cm.

| Family | Species | length | SMR | MMR | FMR | FAS | FSA |
| --- | --- | --- | --- | --- | --- | --- | --- |
| Acanthuridae | *Ctenochaetus striatus* | 10 | 0.044 | 0.111 | 0.065 | 2.542 | 1.248 |
| Acanthuridae | *Ctenochaetus striatus* | 11 | 0.054 | 0.138 | 0.080 | 2.550 | 1.241 |
| Acanthuridae | *Ctenochaetus striatus* | 12 | 0.066 | 0.168 | 0.097 | 2.555 | 1.235 |
| Acanthuridae | *Ctenochaetus striatus* | 13 | 0.079 | 0.202 | 0.115 | 2.562 | 1.229 |
| Acanthuridae | *Ctenochaetus striatus* | 14 | 0.094 | 0.239 | 0.136 | 2.568 | 1.224 |
| Acanthuridae | *Ctenochaetus striatus* | 15 | 0.109 | 0.280 | 0.157 | 2.571 | 1.219 |
| Acanthuridae | *Ctenochaetus striatus* | 16 | 0.127 | 0.325 | 0.181 | 2.573 | 1.215 |
| Acanthuridae | *Ctenochaetus striatus* | 17 | 0.145 | 0.373 | 0.207 | 2.583 | 1.211 |
| Acanthuridae | *Ctenochaetus striatus* | 18 | 0.165 | 0.425 | 0.234 | 2.589 | 1.207 |
| Acanthuridae | *Ctenochaetus striatus* | 19 | 0.187 | 0.481 | 0.263 | 2.589 | 1.204 |
| Acanthuridae | *Ctenochaetus striatus* | 20 | 0.210 | 0.541 | 0.294 | 2.593 | 1.201 |
| Acanthuridae | *Ctenochaetus striatus* | 21 | 0.234 | 0.606 | 0.327 | 2.603 | 1.199 |
| Acanthuridae | *Ctenochaetus striatus* | 22 | 0.260 | 0.674 | 0.362 | 2.608 | 1.196 |
| Acanthuridae | *Ctenochaetus striatus* | 23 | 0.287 | 0.746 | 0.399 | 2.605 | 1.194 |
| Acanthuridae | *Ctenochaetus striatus* | 24 | 0.316 | 0.823 | 0.438 | 2.612 | 1.192 |
| Acanthuridae | *Naso lituratus* | 17 | 0.127 | 0.576 | 0.204 | 4.591 | 1.302 |
| Acanthuridae | *Naso lituratus* | 18 | 0.144 | 0.657 | 0.235 | 4.647 | 1.316 |
| Acanthuridae | *Naso lituratus* | 19 | 0.161 | 0.744 | 0.269 | 4.683 | 1.331 |
| Acanthuridae | *Naso lituratus* | 20 | 0.180 | 0.836 | 0.306 | 4.710 | 1.347 |
| Acanthuridae | *Naso lituratus* | 21 | 0.200 | 0.932 | 0.346 | 4.763 | 1.363 |
| Acanthuridae | *Naso lituratus* | 22 | 0.222 | 1.039 | 0.389 | 4.797 | 1.378 |
| Acanthuridae | *Naso lituratus* | 23 | 0.244 | 1.150 | 0.435 | 4.829 | 1.393 |
| Acanthuridae | *Naso lituratus* | 24 | 0.267 | 1.268 | 0.485 | 4.858 | 1.408 |
| Acanthuridae | *Naso lituratus* | 25 | 0.292 | 1.393 | 0.538 | 4.890 | 1.423 |
| Acanthuridae | *Naso lituratus* | 26 | 0.317 | 1.525 | 0.595 | 4.921 | 1.438 |
| Acanthuridae | *Naso lituratus* | 27 | 0.344 | 1.663 | 0.655 | 4.962 | 1.453 |
| Acanthuridae | *Naso lituratus* | 28 | 0.372 | 1.805 | 0.721 | 4.989 | 1.470 |
| Acanthuridae | *Naso lituratus* | 29 | 0.401 | 1.959 | 0.789 | 5.023 | 1.484 |
| Acanthuridae | *Naso lituratus* | 30 | 0.431 | 2.120 | 0.861 | 5.058 | 1.499 |
| Acanthuridae | *Zebrasoma scopas* | 8 | 0.022 | 0.055 | 0.030 | 2.479 | 1.176 |
| Acanthuridae | *Zebrasoma scopas* | 9 | 0.029 | 0.072 | 0.039 | 2.528 | 1.189 |
| Acanthuridae | *Zebrasoma scopas* | 10 | 0.036 | 0.092 | 0.050 | 2.582 | 1.202 |
| Acanthuridae | *Zebrasoma scopas* | 11 | 0.043 | 0.114 | 0.062 | 2.630 | 1.214 |
| Acanthuridae | *Zebrasoma scopas* | 12 | 0.052 | 0.139 | 0.076 | 2.675 | 1.226 |
| Acanthuridae | *Zebrasoma scopas* | 13 | 0.062 | 0.167 | 0.091 | 2.717 | 1.237 |
| Acanthuridae | *Zebrasoma scopas* | 14 | 0.072 | 0.198 | 0.108 | 2.755 | 1.249 |
| Acanthuridae | *Zebrasoma scopas* | 15 | 0.083 | 0.232 | 0.126 | 2.792 | 1.260 |
| Acanthuridae | *Zebrasoma scopas* | 16 | 0.095 | 0.269 | 0.147 | 2.829 | 1.270 |
| Acanthuridae | *Zebrasoma scopas* | 17 | 0.108 | 0.309 | 0.169 | 2.859 | 1.281 |
| Acanthuridae | *Zebrasoma scopas* | 18 | 0.122 | 0.352 | 0.193 | 2.889 | 1.291 |
| Balistidae | *Odonus niger* | 10 | 0.029 | 0.174 | 0.083 | 5.922 | 1.905 |
| Balistidae | *Odonus niger* | 11 | 0.036 | 0.217 | 0.106 | 6.061 | 1.977 |
| Balistidae | *Odonus niger* | 12 | 0.043 | 0.265 | 0.133 | 6.164 | 2.048 |
| Balistidae | *Odonus niger* | 13 | 0.051 | 0.317 | 0.164 | 6.295 | 2.113 |
| Balistidae | *Odonus niger* | 14 | 0.059 | 0.376 | 0.200 | 6.422 | 2.185 |
| Balistidae | *Odonus niger* | 15 | 0.068 | 0.439 | 0.240 | 6.502 | 2.250 |
| Balistidae | *Odonus niger* | 16 | 0.078 | 0.509 | 0.285 | 6.589 | 2.321 |
| Balistidae | *Odonus niger* | 17 | 0.088 | 0.585 | 0.335 | 6.650 | 2.388 |
| Balistidae | *Odonus niger* | 18 | 0.099 | 0.667 | 0.391 | 6.729 | 2.459 |
| Balistidae | *Odonus niger* | 19 | 0.111 | 0.754 | 0.452 | 6.812 | 2.526 |
| Balistidae | *Odonus niger* | 20 | 0.123 | 0.846 | 0.520 | 6.902 | 2.599 |
| Balistidae | *Odonus niger* | 21 | 0.136 | 0.944 | 0.595 | 6.974 | 2.672 |
| Balistidae | *Odonus niger* | 22 | 0.149 | 1.050 | 0.674 | 7.058 | 2.736 |
| Balistidae | *Odonus niger* | 23 | 0.164 | 1.163 | 0.763 | 7.143 | 2.809 |
| Balistidae | *Odonus niger* | 24 | 0.179 | 1.279 | 0.858 | 7.211 | 2.875 |
| Balistidae | *Odonus niger* | 25 | 0.194 | 1.404 | 0.961 | 7.270 | 2.942 |
| Balistidae | *Odonus niger* | 26 | 0.211 | 1.534 | 1.074 | 7.342 | 3.019 |
| Chaetodontidae | *Chaetodon ornatissimus* | 9 | 0.027 | 0.079 | 0.039 | 2.965 | 1.231 |
| Chaetodontidae | *Chaetodon ornatissimus* | 10 | 0.033 | 0.102 | 0.050 | 3.039 | 1.255 |
| Chaetodontidae | *Chaetodon ornatissimus* | 11 | 0.041 | 0.127 | 0.064 | 3.101 | 1.281 |
| Chaetodontidae | *Chaetodon ornatissimus* | 12 | 0.049 | 0.155 | 0.078 | 3.160 | 1.304 |
| Chaetodontidae | *Chaetodon ornatissimus* | 13 | 0.058 | 0.186 | 0.095 | 3.214 | 1.325 |
| Chaetodontidae | *Chaetodon ornatissimus* | 14 | 0.068 | 0.221 | 0.114 | 3.268 | 1.347 |
| Chaetodontidae | *Chaetodon ornatissimus* | 15 | 0.078 | 0.260 | 0.135 | 3.325 | 1.369 |
| Chaetodontidae | *Chaetodon ornatissimus* | 16 | 0.090 | 0.301 | 0.159 | 3.377 | 1.391 |
| Chaetodontidae | *Chaetodon ornatissimus* | 17 | 0.102 | 0.346 | 0.185 | 3.418 | 1.414 |
| Chaetodontidae | *Chaetodon ornatissimus* | 18 | 0.114 | 0.395 | 0.213 | 3.456 | 1.436 |
| Chaetodontidae | *Chaetodon ornatissimus* | 19 | 0.128 | 0.448 | 0.244 | 3.503 | 1.456 |
| Serranidae | *Cephalopholis argus* | 12 | 0.028 | 0.142 | 0.074 | 5.104 | 1.841 |
| Serranidae | *Cephalopholis argus* | 13 | 0.033 | 0.171 | 0.088 | 5.207 | 1.851 |
| Serranidae | *Cephalopholis argus* | 14 | 0.038 | 0.204 | 0.104 | 5.326 | 1.861 |
| Serranidae | *Cephalopholis argus* | 15 | 0.044 | 0.239 | 0.120 | 5.433 | 1.869 |
| Serranidae | *Cephalopholis argus* | 16 | 0.050 | 0.279 | 0.138 | 5.534 | 1.880 |
| Serranidae | *Cephalopholis argus* | 17 | 0.057 | 0.322 | 0.158 | 5.641 | 1.884 |
| Serranidae | *Cephalopholis argus* | 18 | 0.064 | 0.368 | 0.179 | 5.752 | 1.892 |
| Serranidae | *Cephalopholis argus* | 19 | 0.072 | 0.418 | 0.201 | 5.839 | 1.898 |
| Serranidae | *Cephalopholis argus* | 20 | 0.080 | 0.472 | 0.224 | 5.919 | 1.904 |
| Serranidae | *Cephalopholis argus* | 21 | 0.088 | 0.529 | 0.250 | 6.007 | 1.910 |
| Serranidae | *Cephalopholis argus* | 22 | 0.098 | 0.590 | 0.276 | 6.087 | 1.912 |
| Serranidae | *Cephalopholis argus* | 23 | 0.107 | 0.655 | 0.304 | 6.157 | 1.919 |
| Serranidae | *Cephalopholis argus* | 24 | 0.117 | 0.726 | 0.333 | 6.224 | 1.924 |
| Serranidae | *Cephalopholis argus* | 25 | 0.127 | 0.801 | 0.363 | 6.304 | 1.927 |
| Serranidae | *Cephalopholis argus* | 26 | 0.138 | 0.878 | 0.394 | 6.379 | 1.928 |
| Serranidae | *Cephalopholis argus* | 27 | 0.149 | 0.962 | 0.427 | 6.449 | 1.929 |
| Serranidae | *Cephalopholis argus* | 28 | 0.160 | 1.046 | 0.461 | 6.512 | 1.931 |
| Serranidae | *Cephalopholis argus* | 29 | 0.172 | 1.137 | 0.497 | 6.558 | 1.934 |
| Serranidae | *Cephalopholis argus* | 30 | 0.185 | 1.233 | 0.535 | 6.628 | 1.938 |
| Serranidae | *Cephalopholis argus* | 31 | 0.198 | 1.332 | 0.572 | 6.694 | 1.938 |
| Serranidae | *Cephalopholis argus* | 32 | 0.211 | 1.434 | 0.613 | 6.764 | 1.941 |
| Serranidae | *Cephalopholis argus* | 33 | 0.225 | 1.542 | 0.654 | 6.833 | 1.942 |
| Serranidae | *Cephalopholis argus* | 34 | 0.240 | 1.655 | 0.697 | 6.880 | 1.946 |
| Serranidae | *Cephalopholis argus* | 35 | 0.254 | 1.770 | 0.743 | 6.943 | 1.950 |
| Serranidae | *Cephalopholis argus* | 36 | 0.269 | 1.892 | 0.791 | 7.006 | 1.955 |
| Serranidae | *Cephalopholis argus* | 37 | 0.285 | 2.018 | 0.839 | 7.065 | 1.959 |

1.
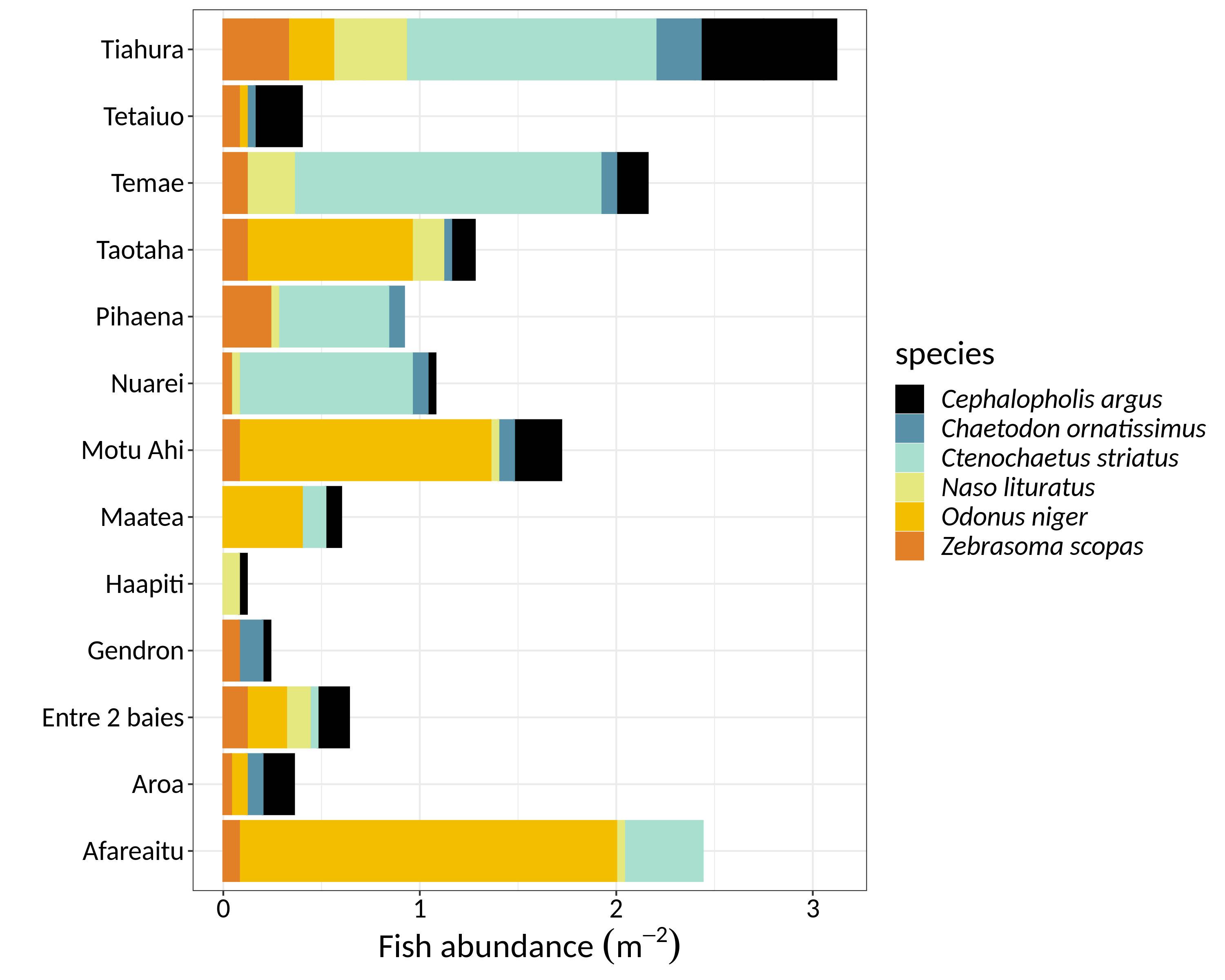

   1. **Figure S5.** Fish abundance (m^-2^) of the studied sites. Each color represents the abundance of a specific studied reef fish species.
